# Mitochondrial Superoxide Signaling Contributes to Norepinephrine-Mediated T-Lymphocyte Cytokine Profiles

**DOI:** 10.1371/journal.pone.0164609

**Published:** 2016-10-11

**Authors:** Adam J. Case, Colton T. Roessner, Jun Tian, Matthew C. Zimmerman

**Affiliations:** Department of Cellular and Integrative Physiology, University of Nebraska Medical Center, Omaha, NE, United States of America; University of Illinois at Chicago, UNITED STATES

## Abstract

Norepinephrine (NE) produces multifaceted regulatory patterns in T-lymphocytes. Recently, we have shown that NE utilizes redox signaling as evidenced by increased superoxide (O_2_^●-^) causally linked to the observed changes in these cells; however, the source of this reactive oxygen species (ROS) remains elusive. Herein, we hypothesized that the source of increased O_2_^●-^ in NE-stimulated T-lymphocytes is due to disruption of mitochondrial bioenergetics. To address this hypothesis, we utilized purified mouse splenic CD4+ and CD8+ T-lymphocytes stimulated with NE and assessed O_2_^●-^ levels, mitochondrial metabolism, cellular proliferation, and cytokine profiles. We demonstrate that the increase in O_2_^●-^ levels in response to NE is time-dependent and occurs at later points of T-lymphocyte activation. Moreover, the source of O_2_^●-^ was indeed the mitochondria as evidenced by enhanced MitoSOX Red oxidation as well as abrogation of this signal by the addition of the mitochondrial-targeted O_2_^●-^-scavenging antioxidant MitoTempol. NE-stimulated T-lymphocytes also demonstrated decreased mitochondrial respiratory capacity, which suggests disruption of mitochondrial metabolism and the potential source of increased mitochondrial O_2_^●-^. The effects of NE in regards to redox signaling appear to be adrenergic receptor-dependent as specific receptor antagonists could reverse the increase in O_2_^●-^; however, differential receptors regulating these processes were observed in CD4+ versus CD8+ T-lymphocytes. Finally, mitochondrial O_2_^●-^ was shown to be mechanistic to the NE-mediated T-lymphocyte phenotype as supplementation of MitoTempol could reverse specific changes in cytokine expression observed with NE treatment. Overall, these studies indicate that mitochondrial metabolism and O_2_^●-^-mediated redox signaling play a regulatory role in the T-lymphocyte response to NE.

## Introduction

Enhanced activation of the sympathetic nervous system is associated with numerous pathological conditions ranging from hypertension, heart failure, diabetes, and even psychological stress[1–4]. Sympathoexcitation leads to increased norepinephrine (NE) outflow to peripheral organs including the predominantly sympathetic-innervated lymphoid organs including the bone marrow, lymph nodes, and spleen[5]. Resident immune cells in these lymphoid organs have been shown to possess adrenergic receptors[6, 7], and over the last four decades it has become well-accepted that autonomic regulation of the immune system is a tangible phenomenon[8, 9]. However, catecholamines appear to elicit a complex pattern of regulation on immune cells dependent upon numerous variables including cell type, activation status, polarization and differentiation, organ of residence, and many others[10, 11].

One immune cell type that has been extensively studied for its effects with NE is T-lymphocytes. Early *in vitro* work with T-lymphocytes demonstrated that NE slows the proliferation of these cells and decreases the amount of pro-inflammatory cytokine production through an inhibition of interleukin 2 (IL-2), and this observation has been validated by numerous laboratories utilizing various populations of T-lymphocytes[11–15]. Moreover, NE appears to produce this inhibitory effect primarily through a β2 adrenergic receptor-mediated mechanism[14]. In contrast, several investigations have shown that NE may enhance the pro-inflammatory state of T-lymphocytes particularly in regards to interferon gamma (IFNγ) production or in the ability to fight infection[16–18]. Additionally, other studies have identified NE-mediated effects on T-lymphocytes that are initiated via adrenergic receptors other than just the β2 isoform[15, 19–21]. Taken together, the complexity and disparity of observations in regards to NE-mediated effects on T-lymphocytes suggests the potential for multidimensional regulatory mechanisms that are not yet fully understood.

In 2013, Fadel and colleagues observed that human peripheral blood mononuclear cells produced increased reactive oxygen species (ROS), particularly superoxide (O_2_^●-^), in response to NE, and further suggested this to be an α2-adrenergic receptor-mediated effect[19]. We have recently confirmed and extended these findings specifically in T-lymphocytes both *in vivo* and *in vitro* in a mouse model of sympathoexcitation[22]. Our results additionally suggested this increase in O_2_^●-^ was causal to the NE-driven effects in the T-lymphocytes as O_2_^●-^-scavenging antioxidant supplementation was able to partially inhibit the NE-mediated T-lymphocyte phenotype[22]. To our knowledge, these findings were the first to report the potential for redox signaling in the regulation of NE-mediated effects in T-lymphocytes.

In the work presented herein, we aimed to expand upon our previous observation and identify the source of O_2_^●-^ in NE-stimulated T-lymphocytes. Due to our previous observations that the O_2_^●-^ produced in response to NE appeared to be time dependent, we hypothesized that NE may be altering mitochondrial metabolism and in turn affecting mitochondrial-derived O_2_^●-^ specifically. We and others have recently reported that metabolism and redox signaling play an integral role in T-lymphocyte activation, polarization, and function[23–28], and here we demonstrate that NE may affect these central processes adding to the complexity of catecholaminergic regulation of T-lymphocytes.

## Materials and Methods

### Mice

C57BL/6 breeding pair mice were originally purchased from Envigo RMS (Indianapolis, IN). All experiments were performed using progeny virgin male mice (Age 8–12 weeks, weight 20–25 g) kept in group/social housing from time of weaning. Breeding colonies were established and maintained for a minimum of 6 months within one housing room to eliminate shipping or room change stressors[29, 30]. With the exception of biweekly cage changes by a single female technician[31], mice were not handled until sacrifice for T-lymphocyte isolation. Mice were given access to standard chow (Teklad Laboratory Diet #7012, Envigo RMS, Indianapolis, IN) and water *ad libitum*. Mice were euthanized by pentobarbital overdose (150 mg/kg, Fatal Plus, Vortech Pharmaceuticals, Dearborn, MI) administered intraperitoneally. All procedures were reviewed and approved by the University of Nebraska Medical Center Institutional Animal Care and Use Committee.

### T-lymphocyte isolation

Spleens from mice were dissected and physically disrupted into single cell suspensions using ground glass slides (Thermo Fisher Scientific #6684h61, Waltham, MA), and run through 70 μM nylon mesh filters (Thermo Fisher Scientific #352350, Waltham, MA) to remove large tissue debris. Red blood cell lysis buffer (15.5 mM NH_4_Cl, 1 mM KHCO_3_, 10 μM EDTA) was used to remove contaminating erythrocytes. After a second filtering through 70 μM nylon mesh, CD4+ and CD8+ T-lymphocytes were isolated by negative selection using the EasySep^™^ Mouse CD4+ T-Cell Isolation Kit (StemCell Technologies #19852, Vancouver, BC) or CD8+ T-Cell Isolation Kit (StemCell Technologies # 19853, Vancouver, BC), respectively. The purity of the respective T-lymphocyte populations was assessed by flow cytometry at >95%.

### Treatment regimens

For treatment during T-lymphocyte activation, T-lymphocytes were seeded on tissue culture plates coated using 10 μg/mL anti-CD3ε antibody (eBioscience clone 145-2C11, #16-0031-086, San Diego, CA) and 1 μg/mL anti-CD28 antibody (eBioscience clone 37.51, #16-0281-85, San Diego, CA) as this concentration has been validated by our laboratory previously to provide sufficient stimulation for both CD4+ and CD8+ T-lymphocytes grown independently[22]. T-lymphocytes were cultured using RPMI Medium 1640 without phenol red (Gibco #11835–030, Grand Island, NY) supplemented with 10% fetal bovine serum (Atlanta Biologicals #S11150, Lawrenceville, GA), 2 mM L-glutamine (Hyclone #SH30034.02, Waltham, MA), 10 mM HEPES (Thermo Fisher Scientific #BP299, Waltham, MA), 1% Penicillin/Streptomycin (Gibco #15140–122, Grand Island, NY), and 50 μM β-mercaptoethanol (Sigma-Aldrich #M6250, St. Louis, MO). Doses ranging from 0.1–100 μM NE (Sigma-Aldrich #A7256, St. Louis, MO), prazosin (Sigma-Aldrich #P7791, St. Louis, MO), atipamezole (Sigma-Aldrich #A9611, St. Louis, MO), metoprolol (Sigma-Aldrich #M5391, St. Louis, MO), ICI 118,511 (Sigma-Aldrich #I127, St. Louis, MO), atomoxetine (Sigma-Aldrich #Y0001586, St. Louis, MO), or vehicle (phosphate-buffered saline, PBS) were added at time of plating and replenished every 24 hours for the duration of the experiment to counteract degradation by endogenous T-lymphocyte monoamine oxidases[32]. A concentration of 1 μM was universally used for follow-up analyses due to its optimal effects with minimal toxicity, as well as previous work utilizing this established physiological dose[33–35]. At time of analysis, cells were harvested and counted utilizing size exclusion for live cells on a Beckman Coulter counter. Cell media was used for cytokine analysis. For acute treatment, freshly isolated T-lymphocytes were treated with 1 μM NE for 30 minutes prior to respective analysis.

### Immunophenotyping

After culture, T-lymphocytes were counted and resuspended in pre-warmed fresh phenol red-free culture media (see aforementioned media recipe). Cells were blocked using anti-CD16/CD32 antibody (BD Biosciences clone 2.4G2, #553141, San Jose, CA) prior to staining. The following antibodies were used at 1:100 to identify naïve, effector, or memory lineages of T-lymphocytes: APC-CD44 (BD Biosciences clone IM7, # 561862, San Jose, CA) and BV421-CD62L (BD Biosciences clone MEL-14, # 562910, San Jose, CA). Antibodies were added and incubated for 30 min at 37°C, and then washed twice using pre-warmed fresh media. T-lymphocytes were immediately analyzed by flow cytometry on a LSRII flow cytometer (Becton Dickinson, Franklin Lakes, NJ) and quantified using FlowJo cytometric analysis software (Tree Star, Ashland, OR). Cell lineages were defined as follows: Naïve, CD62L+, CD44-; Effector/Effector Memory, CD62L-, CD44+; Central Memory, CD62L+, CD44+. Immunophenotyping was performed in warmed media due to concurrent superoxide measurements (see below).

### Superoxide measurement

Total cellular O_2_^●-^ levels were quantified by flow cytometry utilizing dihydroethidium (DHE) and mitochondrial-derived O_2_^●-^ levels were measured using MitoSOX Red (DHE conjugated to a mitochondrial localization tag)[22, 25]. T-lymphocytes were resuspended in phenol red-free culture media (see aforementioned media recipe) with 10 μM DHE (VWR #101447–534, Chicago, IL) or MitoSOX Red (Thermo Fisher Scientific #M36008, Waltham, MA) for 30 min at 37°C. Cells were analyzed on a FACSCalibur or LSRII flow cytometers at 488 nm excitation and 575 nm emission. Electron paramagnetic resonance (EPR) spectroscopy was also utilized for detection of intracellular O_2_^●-^ as previously described[36]. Briefly, T-lymphocytes were incubated for 30 minutes at 37°C with the cell-permeable O_2_^●-^-sensitive spin probe 1-hydroxy-3-methoxycarbonyl-2,2,5,5-tetramethylpyrrolidine (CMH, 200 μmol/L, Noxygen Science Transfer and Diagnostics, Elzach, Germany) in a Krebs-HEPES buffer (pH 7.4) containing (in mmol/L): 99 NaCl, 4.69 KCl, 2.5 CaCl_2_, 1.2 MgSO_4_, 25 NaHCO_3_, 1.03 KH_2_PO_4_, 5.6 D-glucose, 20 HEPES, and supplemented with the metal chelators DETC (5 μM) and deferoxamine (25 μM). Cells were analyzed using a Bruker e-scan EPR spectrometer. The following EPR settings were used: field sweep width, 60.0 gauss; microwave frequency, 9.75 kHz; microwave power, 21.90 mW; modulation amplitude, 2.37 gauss; conversion time, 10.24 ms; time constant, 40.96 ms. For inhibition of NADPH oxidases, 1 μM of the NADPH oxidase/flavoprotein inhibitor diphenyleneiodonium (DPI; Sigma-Aldrich #D2926, St. Louis, MO) was added to cell cultures 1 hour prior to harvest and analysis. To scavenge total or mitochondrial-specific O_2_^●-^, 1 μM of Tempol (Enzo Life Sciences #ALX-430-081, Farmingdale, NY) or MitoTempol (Enzo Life Sciences #ALX-430-150, Farmingdale, NY) was added, respectively, at time of plating and replenished every 24 hours for the duration of the experiment.

### Mitochondrial bioenergetics analysis

To measure T-lymphocyte mitochondrial bioenergetics, a Seahorse Bioscience XFp extracellular flux analyzer was used. This device utilizes specialized microplates to create a closed chamber able measure real-time oxygen consumption by mitochondria in live cells exposed to various stimuli through multiple designed injection ports. Optimal seeding density of T-lymphocytes was established at 200,000 cells per well and was utilized for all experiments. Additionally, mitochondrial agents (Seahorse Bioscience Cell Mito Stress Test Kit #103015–100, Boston, MA) were pre-optimized at 1 μM oligomycin, 1 μM FCCP, and 10 μM rotenone/antimycin A to elicit maximal effects on mitochondrial respiration. Microplates were pretreated with 1 μg/cm^2^ Cell-Tak (Corning #354240, Corning, NY) to allow T-lymphocyte adhesion to the chamber. For acute NE treatment, T-lymphocytes were freshly isolated, counted, and immediately seeded on microplates in minimal DMEM (Seahorse Bioscience XF Base Medium #102353, Boston, MA) supplemented with 11 mM D-glucose and 2 mM L-glutamine. NE was administered by way of Seahorse injection port and cells analyzed for 30 minutes prior to mitochondrial stress test. For treatment of NE in activated T-lymphocytes, T-lymphocytes were cultured for 96 hours as previously described in the presence of 1 μM NE. Cells were harvested, counted, and seeded on microplates in minimal DMEM supplemented with 11 mM D-glucose and 2 mM L-glutamine. Mitochondrial stress test was performed immediately without any additional NE administration. Cells from one mouse (vehicle and NE treated) were run on a single plate in technical replicates; biological replicate plates were run and data pooled for analysis.

### Cytokine analysis

Extracellular secreted cytokine analysis was performed on cell media from T-lymphocytes cultured for 96 hours. Analysis was performed using the mouse Th1/Th2/Th17 cytometric bead array (BD Biosciences #560485, San Jose, CA) as per manufacturer’s instructions. Briefly, media was combined with antibody-coated fluorescent beads for 7 specific cytokines used to profile various T-lymphocyte subtypes. Beads were analyzed on a LSRII flow cytometer for quantification of specific cytokines.

### Statistics

Data are presented as mean ± standard error of the mean (SEM). For two group comparison, significance was assessed using the paired Student’s t-test. For multiple group comparison, significance was assessed using 2-way ANOVA followed by Bonferroni post-hoc analysis. Differences were considered significant at p<0.05.

## Results

### NE increases O_2_^●-^ in both CD4+ and CD8+ T-lymphocytes

**Fig 1:**
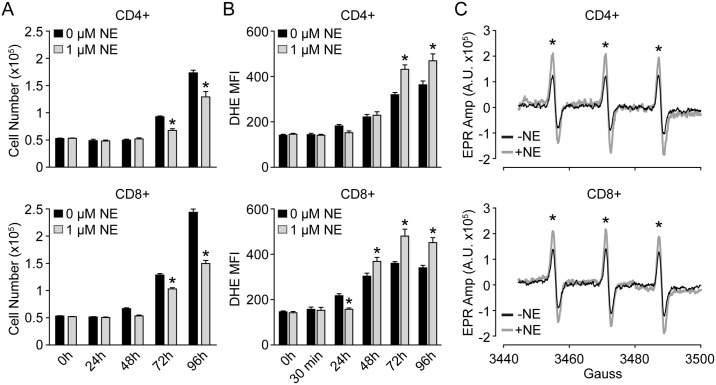
NE provokes inverse effects on T-lymphocyte growth and O_2_^●-^ levels. T-lymphocytes were isolated, purified, and activated via CD3/CD28 stimulation in the presence of 1 μM NE. **A**. T-lymphocyte growth curves at various time points of ex vivo culture. N = 4. **B**. Quantification of DHE oxidation in CD4+ and CD8+ T-lymphocytes at various time points post-activation. N = 4. **C**. Representative EPR spectra showing amplitude (Amp) for T-lymphocytes stimulated in the presence or absence of 1 μM NE for 96 hours. N = 3. *p<0.05 vs. 0 μM NE by Student’s t-test at respective time points.

In our previous report, we demonstrated that NE elevates steady-state O_2_^●-^ levels in unfractionated T-lymphocytes isolated from a mouse model of sympathoexcitation[22]. To expand upon this observation, we isolated and purified primary murine CD4+ or CD8+ T-lymphocytes and cultured them separately in the presence of increasing amounts of NE for 96 hours. First, both CD4+ and CD8+ T-lymphocytes demonstrated a dose-dependent decrease in cell proliferation (identified previously as a defect in proliferation as opposed to increased apoptosis[22]) with increasing amounts of NE (Fig 1A, S1A Fig). Moreover, the decrease in proliferation was correlated with an increase in cellular O_2_^●-^ levels as evidenced by increased DHE oxidation and EPR spectrum amplitude in both CD4+ and CD8+ T-lymphocytes primarily at later time points of activation (Fig 1B and 1C, S1B Fig). Additionally, the observed whole-population shift of DHE oxidation (S1B Fig) implies the increase in O_2_^●-^ is a pan-T-lymphocyte phenomenon and not specific to one specific subtype of differentiated cells. This interpretation is further supported by data demonstrating that all NE-treated T-lymphocytes in different stages of activation (*i*.*e*. naïve, central memory, and effector/effector memory) have increases in O_2_^●-^ compared to control cells even though NE aberrantly affects the percentages of T-lymphocytes in these respective populations (S2 Fig). Last, a dose of 1 μM NE was utilized for the remainder of the experiments as it was the lowest dose observed to decrease cellular proliferation and increase O_2_^●-^ levels, as well as has been previously reported as a physiologically relevant concentration[11, 34, 35, 37].

### Mitochondria are the source of O_2_^●-^ in NE-stimulated T-lymphocytes

**Fig 2:**
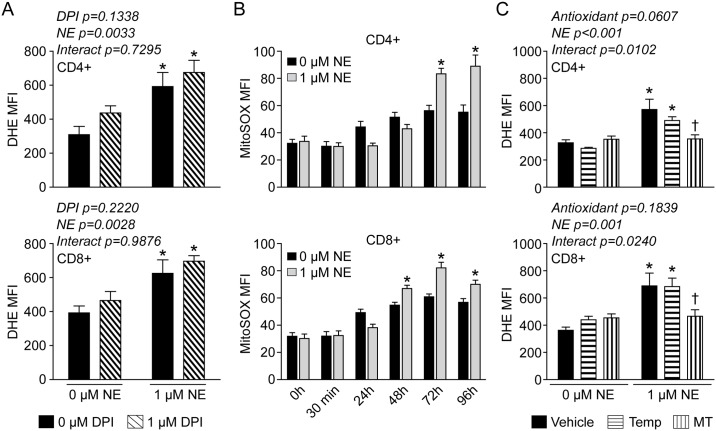
Mitochondrial O_2_^●-^ is increased in T-lymphocytes treated with NE. T-lymphocytes were isolated, purified, and activated via CD3/CD28 stimulation in the presence of 1 μM NE. **A**. Quantification of DHE oxidation in CD4+ and CD8+ T-lymphocytes 96 hours post-activation. Cells were incubated with DPI 1 hour prior to and during the incubation with DHE. N = 3. *p<0.05 vs. vehicle with 0 μM NE by 2-way ANOVA followed by Bonferroni post-hoc analysis. **B**. Quantification of MitoSOX red oxidation in CD4+ and CD8+ T-lymphocytes at various time points post-activation. N = 6. *p<0.05 vs. 0 μM NE by Student’s t-test at respective time points. **C**. Quantification of DHE oxidation in CD4+ and CD8+ T-lymphocytes at 96 hours post-activation. 1 μM Tempol (Temp) or MitoTempol (MT) were supplemented at time of plating and every 24 hours post-activation. N = 4. *p<0.05 vs. vehicle with 0 μM NE; ^†^p<0.05 vs. vehicle with 1 μM NE by 2-way ANOVA followed by Bonferroni post-hoc analysis.

Under certain conditions, NE is known to generate free radical species independent of a receptor-mediated or cell-dependent mechanism[38–40]. To ensure the increase in DHE oxidation observed with NE-stimulated T-lymphocytes was not due to direct interaction of the NE with DHE, we tested the ability of NE to oxidize DHE in a cell free environment. We found no evidence that NE was directly increasing DHE oxidation (S3 Fig), which suggested a cell-dependent mechanism of O_2_^●-^ generation. The NADPH oxidase (NOX) enzymes are a predominant source of intracellular O_2_^●-^ and are known to be involved in many redox signaling processes[36, 41, 42]. To identify if NOX enzymes were a source of O_2_^●-^ in NE-stimulated T-lymphocytes, we utilized the pan-NOX inhibitor DPI. While we observed the typical increase in O_2_^●-^ in both CD4+ and CD8+ T-lymphocytes activated in the presence of NE, DPI had no effect in attenuating this response suggesting NOX enzymes are not a predominant source of O_2_^●-^ with NE-stimulation in T-lymphocytes (Fig 2A). Following this, we next examined if mitochondria were producing O_2_^●-^ in response to NE utilizing the mitochondrial targeted O_2_^●-^ probe MitoSOX Red. Intriguingly, we observed significant increases in MitoSOX oxidation in response to NE at later time points of T-lymphocyte activation (Fig 2B) that correlated with the previously observed DHE results (Fig 1B). To further examine mitochondrial O_2_^●-^, we utilized the mitochondrial targeted O_2_^●-^ scavenger MitoTempol. Culturing the cells in the presence of MitoTempol, but not the non-mitochondrial targeted version Tempol, was able to completely attenuate the NE-mediated increase in O_2_^●-^ in both CD4+ and CD8+ T-lymphocytes (Fig 2C), thus further implicating mitochondria as a primary source of NE-mediated O_2_^●-^ in T-lymphocytes.

### Mitochondrial bioenergetics are altered by NE in T-lymphocytes

**Fig 3:**
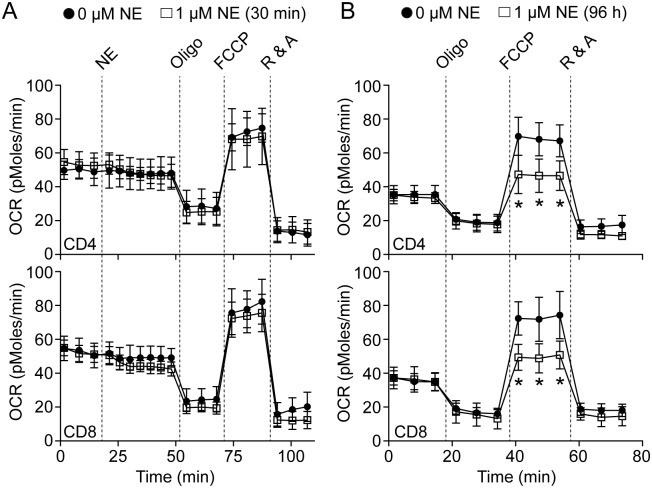
T-lymphocyte mitochondrial reserve respiratory capacity is decreased with NE. T-lymphocytes were isolated and purified. Cells were either immediately analyzed in a Seahorse Bioscience XFp extracellular flux analyzer with a 30-minute acute treatment of 1 μM NE, or activated via CD3/CD28 stimulation in the presence of 1 μM NE for 96 hours prior to analysis. **A**. Cumulative average data of oxygen consumption rate (OCR) measured over-time with acute treatment of NE. N = 9. **B**. Cumulative average data of OCR measured over-time in activated T-lymphocytes cultured in the presence of NE. N = 9. *p<0.05 vs. 0 μM NE by Student’s t-test at respective time points.

Within a mitochondrion, there are many potential sources of O_2_^●-^ generation, but the primary source is due to electron leak onto oxygen from the electron transport chain[43, 44]. Due to this, we measured mitochondrial respiration in NE-stimulated T-lymphocytes utilizing a Seahorse Bioscience extracellular flux bioanalyzer. This technology allows for real-time measurements of mitochondrial metabolism via the measurement of oxygen consumption in response to specific mitochondrial respiratory chain inhibitors[45]. When examining the effects of an acute exposure (30 minutes) of NE to T-lymphocytes, no changes were observed in baseline oxygen consumption or with any mitochondrial inhibitor (Fig 3A), which correlated with the lack of any detectable ROS production at this time point as well (Figs 1B and 2B). In contrast, T-lymphocytes activated in the presence of NE for 96 hours demonstrated a decreased respiratory capacity response to the uncoupling agent carbonilcyanide p-triflouromethoxyphenylhydrazone (FCCP; Fig 3B). Increased ROS production from the mitochondria and decreases in respiratory capacity are often associated, and are additionally correlated with a numerous pathologies suggesting a possible mechanism behind altered T-lymphocyte function in response to NE[46–49]. Moreover, NE does not appear to affect T-lymphocyte mitochondrial ATP levels as the addition of oligomycin demonstrated similar inhibition in both NE and control treated cells (Fig 3B). Overall, these data further support the mitochondria as the source of O_2_^●-^ in NE-treated T-lymphocytes and suggest bioenergetic dysfunction may play a role in the observed phenotype.

### NE-mediated redox signaling appears dependent upon adrenergic receptors

**Fig 4:**
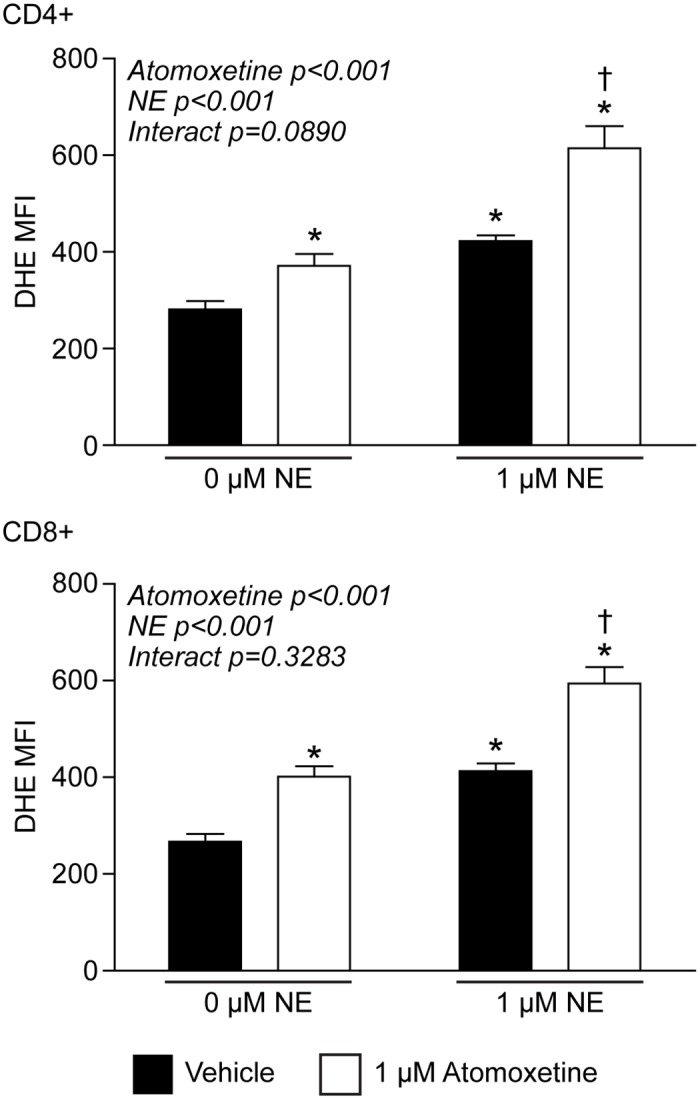
Inhibiting NE transport increases intracellular O_2_^●-^ levels. T-lymphocytes were isolated, purified, and activated via CD3/CD28 stimulation in the presence of NE and/or the norepinephrine transporter inhibitor atomoxetine. Quantification of DHE oxidation in CD4+ (upper) and CD8+ (lower) T-lymphocytes 96 hours post-activation. N = 4. *p<0.05 vs. vehicle with 0 μM NE; ^†^p<0.05 vs. vehicle with 1 μM NE by 2-way ANOVA followed by Bonferroni post-hoc analysis.

We next attempted to understand the mechanism by which NE was causing increased mitochondrial O_2_^●-^ in T-lymphocytes. Aforementioned, NE is able to auto-oxidize to generate free radicals independent of cell or enzyme dependent mechanisms[38–40]. While we demonstrated NE was not directly oxidizing DHE, we postulated that if NE was able to enter the cells it could possibly directly generate O_2_^●-^ intracellularly. However, NE is not cell permeable and requires specific transporters to import the catecholamine across the cell membrane. T-lymphocytes have been shown to express various catecholamine transporters as well as generate their own NE which is transported across the cell membrane and utilized in an autocrine fashion[21, 32, 50–53]. Due to this, we utilized the NE transporter (NET) inhibitor atomoxetine to see if the intracellular import of NE was leading to the increase in O_2_^●-^ in T-lymphocytes. In contrast to our hypothesis, the addition of atomoxetine alone increased O_2_^●-^ within T-lymphocytes, and moreover, produced an additive effect with the supplementation of NE (Fig 4). Additionally, atomoxetine alone demonstrated a dose-dependent decrease in cell number and in combination with NE enhanced the proliferative defect (S4 Fig). Taken together, these data suggest NE transport is not the primary mechanism by which NE mediates intracellular O_2_^●-^ production. Furthermore, the inhibition of NE uptake into the cell appears to enhance the NE-mediated alterations suggesting T-lymphocytes may normally utilize this mechanism to metabolize the catecholamine and limit its effects.

**Fig 5:**
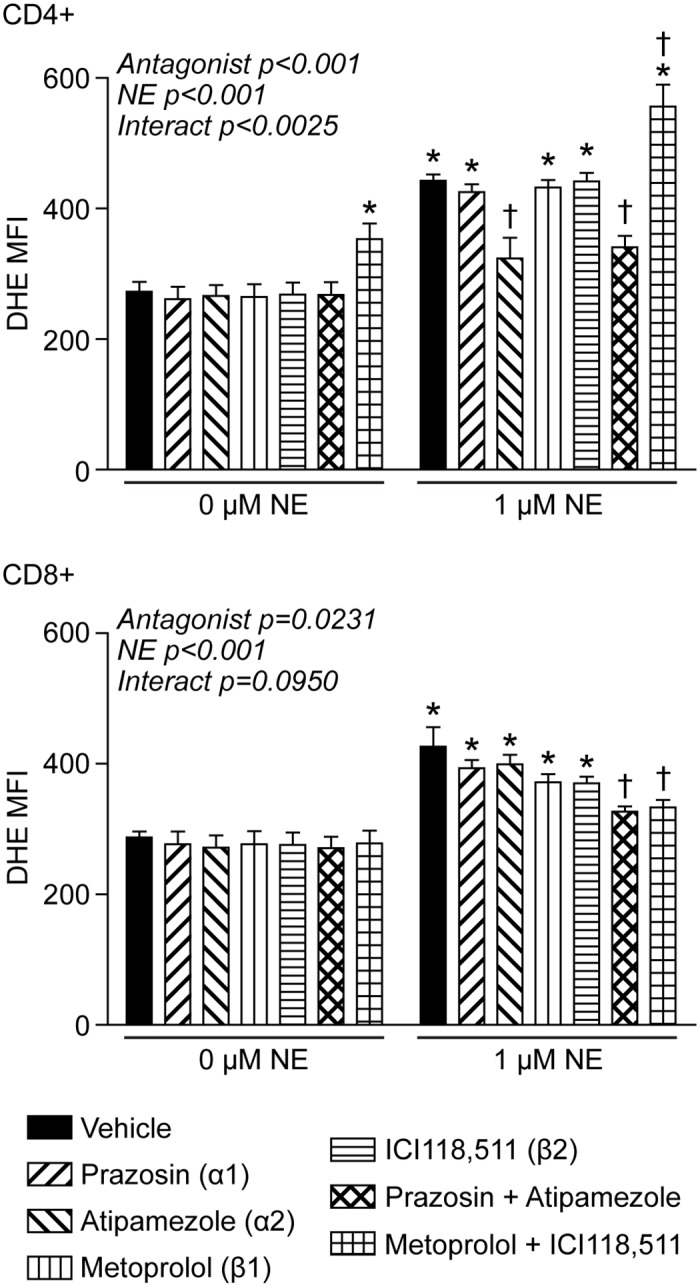
NE-mediated changes in O_2_^●-^ are mediated via various adrenergic receptors. T-lymphocytes were isolated, purified, and cells were activated via CD3/CD28 stimulation in the presence of 1 μM NE or the respective antagonist for 96 hours prior to analysis. Quantification of DHE oxidation in CD4+ (upper) and CD8+ (lower) T-lymphocytes activated in the presence of the treatments for 96 hours. N = 4. *p<0.05 vs. vehicle with 0 μM NE; ^†^p<0.05 vs. vehicle with 1 μM NE by 2-way ANOVA followed by Bonferroni post-hoc analysis.

The observation that inhibition of NE transport increased T-lymphocyte O_2_^●-^ levels suggested that more NE was available to bind extracellular adrenergic receptors, which would potentiate the catecholaminergic effects. To examine the contribution of the adrenergic receptors to the production of NE-mediated O_2_^●-^, we utilized receptor specific antagonists (*i*.*e*. α1, prazosin; α2, atipamezole; β1, metoprolol; β2, ICI 118,511) and measured intracellular O_2_^●-^ levels in T-lymphocytes. While NE significantly increased O_2_^●-^ levels in both CD4+ and CD8+ T-lymphocytes cultured for 96 hours, the specific antagonists demonstrated variable responses in the respective cell types (Fig 5). CD4+ cells showed a significant reduction in NE-driven O_2_^●-^ only when treated with the α2 antagonist (alone or in combination with α1 antagonism). Interestingly, individual β antagonism had no effect, but both β1 and β2 blockade together increased intracellular O_2_^●-^ alone and further exacerbated the increase in O_2_^●-^ when in combination with NE. In contrast, CD8+ cells also showed a trending reduction in NE-driven O_2_^●-^ with every antagonist, but only when combinations were utilized (*i*.*e*. α1 and α2, or β1 and β2) were the results statistically significant. These differential responses between CD4+ and CD8+ cells were not observed with cellular proliferation, as only β-blockade in both cell types demonstrated any significant rescue in cell growth (S5–S7 Figs). Overall, these data suggest that NE mediates intracellular O_2_^●-^ production in T-lymphocytes most likely through the binding and activation of specific adrenergic receptors dependent upon cell type, and moreover, these redox signaling events appear to be discordant to the mechanism driving decreases in T-lymphocyte proliferation.

### NE alters T-lymphocyte cytokine profiles in part due to mitochondrial O_2_^●-^

**Fig 6:**
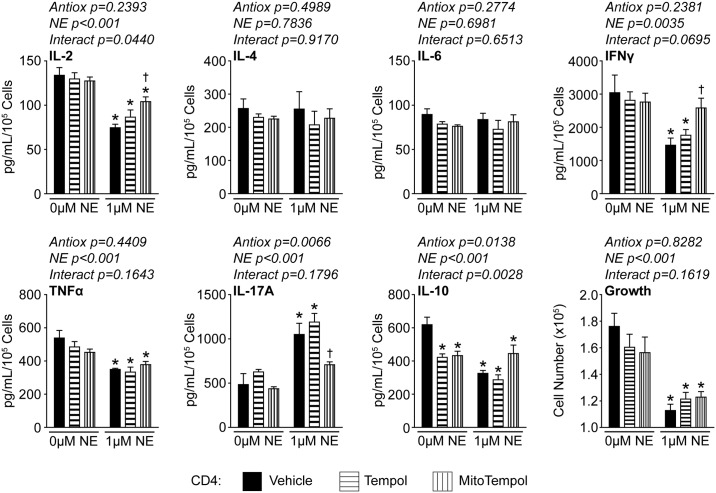
Mitochondrial O_2_^●-^ partially regulates CD4+ T-lymphocyte cytokine profiles. CD4+ T-lymphocytes were isolated, purified, and activated via CD3/CD28 stimulation in the presence of NE, Tempol (1 μM), and/or MitoTempol (1 μM) for 96 hours. Media was harvested for cytokine analysis and results normalized to cell number. N = 5. *p<0.05 vs. vehicle with 0 μM NE; ^†^p<0.05 vs. vehicle with 1 μM NE by 2-way ANOVA followed by Bonferroni post-hoc analysis.

**Fig 7:**
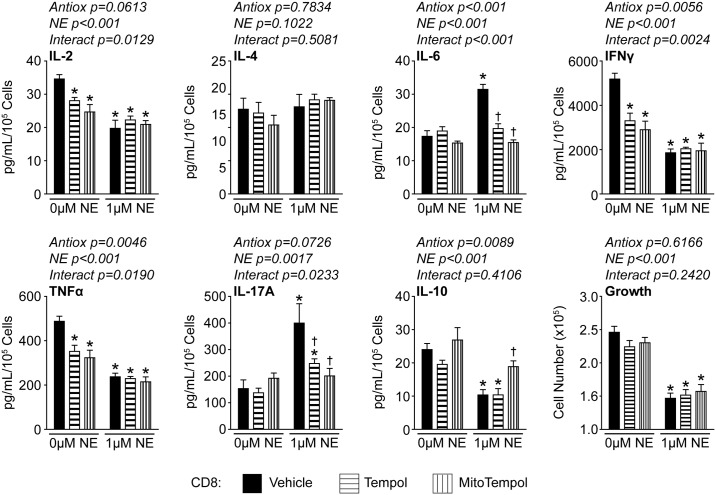
Mitochondrial O_2_^●-^ partially regulates CD8+ T-lymphocyte cytokine profiles. CD8+ T-lymphocytes were isolated, purified, and activated via CD3/CD28 stimulation in the presence of NE, Tempol (1 μM), and/or MitoTempol (1 μM) for 96 hours. Media was harvested for cytokine analysis and results normalized to cell number. N = 5. *p<0.05 vs. vehicle with 0 μM NE; ^†^p<0.05 vs. vehicle with 1 μM NE by 2-way ANOVA followed by Bonferroni post-hoc analysis.

We and others have reported that NE is able to alter cytokine production in T-lymphocytes, however, the specific responses appear to be dependent upon several factors including experimental setup and activation status of the cells[15, 17, 22, 33]. We hypothesized that the changes in cytokine production may be partially mediated by the increase in mitochondrial O_2_^●-^ produced by NE in T-lymphocytes. To address this, we performed cytokine arrays on media from CD4+ or CD8+ T-lymphocytes cultured with NE in the presence or absence of O_2_^●-^ scavenging antioxidants. In CD4+ cells, NE significantly reduced IL-2, IFNγ, tumor necrosis factor α (TNFα), and IL-10 levels, while increasing IL-17A (Fig 6). Interestingly, the addition of MitoTempol was able to significantly restore IL-2, IFNγ, and IL-17A levels in these cells (Fig 6). In CD8+ cells, NE significantly reduced the same four cytokines as CD4+ cells (*i*.*e*. IL-2, IFNγ, TNFα, and IL-10), but also increased both IL-17A and IL-6 levels (Fig 7). In these cells, MitoTempol was able to significantly reestablish IL-6, IL-17A, and IL-10 levels (Fig 7). Interestingly, growth was not restored in either CD4+ or CD8+ T-lymphocytes treated with antioxidants (Figs 6 and 7), which further supports differential regulation between growth and cytokine production in regards to NE-mediated redox signaling. Taken together, these data further support the hypothesis of differential redox regulation in CD4+ and CD8+ cells, but also suggest that increased mitochondrial O_2_^●-^ signaling is only partially contributing to the NE-mediated regulation of T-lymphocytes as not all cytokines or growth could be rescued with the attenuation of the NE-mediated mitochondrial redox signaling.

## Discussion and Conclusion

Investigations into the crosstalk between the nervous and immune systems have been ongoing for several decades. Previously, we observed a new paradigm of redox control in the regulation of T-lymphocytes exposed to NE[22]. In the work presented herein, we have expanded this original observation to identify that mitochondria (and possibly mitochondrial metabolic dysfunction) are the source of the increased O_2_^●-^ in response to NE.

In recent years, it has become appreciated that ROS are necessary for proper T-lymphocyte activation and function. Initial studies identified that both O_2_^●-^ and hydrogen peroxide (H_2_O_2_) were produced upon crosslinking of the T-lymphocyte receptor, which stimulated the ERK signaling pathway modulating T-lymphocyte activation[54]. Soon after this work, T-lymphocytes were identified to possess a distinctly unique NAPDH oxidase that was the source of this ROS upon T-lymphocyte receptor stimulation[55]. Moreover, we and others have shown that mitochondrial derived ROS are also critical in the development and function of T-lymphocytes[23, 25]. However, while ROS have demonstrated a critical functional role in T-lymphocytes, the actual redox signaling mechanisms involved in these processes remain elusive. In the work presented here, we confirm the finding that mitochondrial O_2_^●-^ is increased with T-lymphocyte activation over time and report for the first time that NE-stimulation is able to potentiate this specific ROS production which can alter T-lymphocyte cytokine production. How NE facilitates its effects on the mitochondria remains unclear, but we hypothesize the mediator may be cyclic AMP (cAMP) or its derivatives. Early work examining NE effects on T-lymphocytes demonstrated the observed phenotype is highly attributed to a significant induction of cAMP via the classic G protein-coupled receptor pathway[5, 9]. Recently, it has been observed that cAMP derivatives may affect mitochondrial ROS and metabolism via altering the mitochondrial permeability transition pore (MPTP)[56, 57]. This pathway may explain how NE is able to exert its effects on the mitochondria in T-lymphocytes. Moreover, this mechanism could explain why we observed increases in O_2_^●-^ over time and not acutely, as a buildup of cAMP may be required to significantly alter MPTP function, mitochondrial polarization, and ROS production. Overall, this hypothesis warrants further investigation, and is a focus of current work in our laboratory.

In addition to ROS, cellular metabolism has become accepted as a primary regulator of T-lymphocyte activation and differentiation. Work from Pearce and colleagues has identified that T-lymphocytes shift their metabolic profiles dependent upon activation and differentiation status[26–28, 58]. For example, naïve T-lymphocytes reside in a relatively quiescent state with minimal metabolic needs, however, upon activation to effector T-lymphocytes these cells utilize the Warburg Effect and heavily rely upon glycolysis over mitochondrial oxidative phosphorylation to fulfill their metabolic demand to proliferate and function[28]. In contrast, memory T-lymphocytes significantly enhance their mitochondrial biomass and rely exclusively on oxidative phosphorylation for their function, which is believed to provide a significant advantage to rapidly respond to a secondary immune insult[59–63]. Mitochondrial respiratory capacity is linked to T-lymphocyte memory cell development[61], and furthermore, others have shown that NE does in fact affect memory T-lymphocyte function[34]. Our data presented herein indicates that NE significantly decreases mitochondrial respiratory capacity, and this mechanism may explain the NE-mediated changes in memory T-lymphocyte function previously reported. However, it remains unclear at this time if the NE-mediated mitochondrial dysfunction leads to the increase in mitochondrial O_2_^●-^ or vice versa.

**Fig 8:**
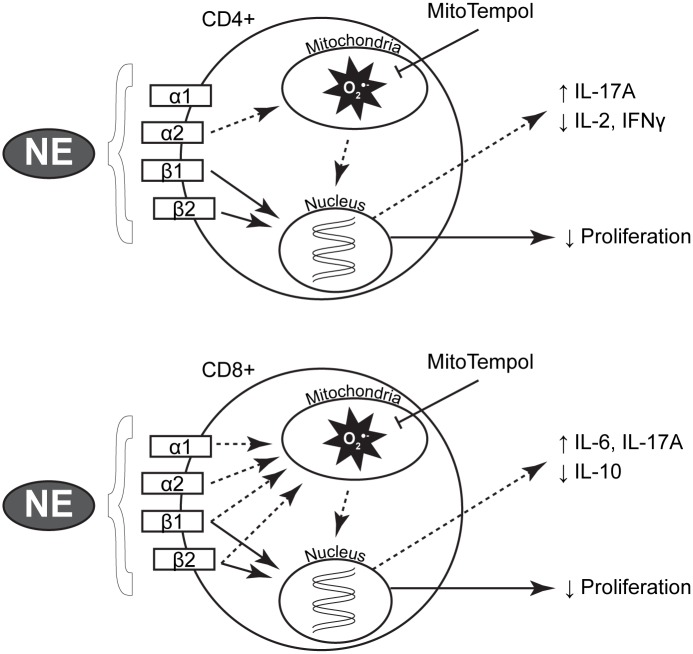
Proposed NE signaling cascade in T-lymphocytes. T-lymphocytes have been shown to express all adrenergic receptors, and NE has binding affinity to each isoform. In both CD4+ and CD8+ cells, NE appears to reduce proliferation via β-receptor canonical signaling (solid arrows) independent of redox events. CD4+ cells utilize the α2 receptor while CD8+ cells use all four adrenergic receptors to initiate redox signaling (dashed arrows) to affect specific subsets of cytokines. In both cells, the addition of MitoTempol can decrease this increase in mitochondrial O_2_^●-^ and reverse the changes in specific cytokine expression.

Our data utilizing mitochondrial-targeted antioxidant supplementation demonstrate a partial rescue of the NE-mediated changes in T-lymphocytes, however, the attenuation of O_2_^●-^ was not able to fully restore growth or all cytokine levels back to normal. These data suggest that these processes are controlled, at least in part, by NE signaling through non-redox regulated mechanisms (*i*.*e*. cAMP and PKA[9, 64], changes in cyclin expression[22], etc.) or that alterations in mitochondrial metabolism may be upstream of the increased O_2_^●-^ production. Thus, attenuating the ROS via O_2_^●-^ scavenging restores the redox-regulated signaling processes but does not eliminate the continued metabolic defect, which may be causal to other aspects of NE-driven changes in T-lymphocytes. For example, metabolite alterations can directly affect the production of specific T-lymphocyte cytokines through regulation of their mRNA via post-transcriptional modifications[65]. Understanding that NE appears to have multifaceted regulation on various cellular processes including metabolism, it is intuitive that the addition of an antioxidant would not be sufficient to reverse all NE-mediated processes in the cells, but only those that are specifically controlled via redox mechanisms (Fig 8).

As part of the phenotype observed in this work, we identified significant changes to cytokine profiles in T-lymphocytes treated with NE. First, we identified that IL-2, IFNγ, and TNFα were all significantly down-regulated in both CD4+ and CD8+ T-lymphocytes treated with NE; a finding that we and others have reported previously[9, 15, 22, 64]. Additionally, we also observed changes in IL-17A, IL-10, and IL-6. These cytokines are not always reportedly altered in T-lymphocytes in response to NE, but we attribute these conflicting results to variances in experimental setup (*i*.*e*. mouse strain, activation stimulus, time course, NE dosage and regimen, etc.) as previously discussed[10, 11]. Interestingly, these three cytokines were all demonstrated to be redox regulated, as antioxidant supplementation could reverse their aberrant expression. Moreover, their pattern of expression (*i*.*e*. increased IL-17A and IL-6, decreased IL-10) suggests a pro-inflammatory profile with increased abundance of T_H_17 polarization. T_H_17 cells primarily produce the pro-inflammatory cytokine IL-17A, and while specific cytokines are known to augment the polarization of T-lymphocytes to this subtype, the intracellular mechanisms controlling this differentiation have yet to be fully elucidated. To date, the major transcription factors known to contribute to T_H_17 differentiation are the retinoic acid receptor-related orphan receptor gamma (RORγ) and Janus kinase/signal transducer and activator of transcription (JAK/STAT) pathways[66]. It may be possible that NE signaling via non-canonical (*i*.*e*. increased mitochondrial O_2_^●-^ or altered cellular metabolites) pathways activates these pro-T_H_17 signaling pathways, thus leading to the redox-regulated increase in IL-17 in T-lymphocytes treated with NE, as reported herein. Interestingly, T_H_17 cells are often associated with autoimmune diseases, and recent reports have demonstrated diseases of increased sympathetic drive appear to be associated with higher abundance of autoimmune and T_H_17 T-lymphocytes[67–69]. For example, patients suffering from post-traumatic stress disorder (PTSD) possess increased incidence rates of the autoimmune disease rheumatoid arthritis[70, 71]. Because PTSD is known to elevate sympathetic drive[72], and that rheumatoid arthritis is also known to be affected by elevated levels of NE[73, 74], it suggests autonomic dysfunction as a possible link between psychological and autoimmune diseases. To date, mitochondrial-targeted antioxidant supplementation has not been examined as a potential treatment in these diseases, and as such our data may support further investigation into the potential of this therapeutic modality.

This study does possess some limitations that will need to be addressed in future work. First, we exclusively utilized CD3/CD28 stimulation as the mechanism to activate T-lymphocytes. While this is an established immunologic procedure, this methodology precludes examination as to whether the redox effects we observed are also produced during antigen-specific activation of T-lymphocytes *in vitro* as well as *in vivo*. These studies along with culturing the T-lymphocytes in polarizing conditions (*i*.*e*. T_H_1, T_H_2, T_reg_) may provide additional information as to how specific subtypes of T-lymphocytes utilize redox regulation in response to NE. Furthermore, our studies were carried out *ex vivo*, in ambient (approximately 21%) oxygen conditions, as well as in the presence of standard high glucose media. Follow-up studies will use *in vivo* models of sympathoexcitation as well as utilize gas controlled work stations to mimic physiological concentrations of oxygen as well as varying metabolic substrates to gain a deeper understanding of the metabolic and redox effects of NE on T-lymphocytes under different physiological conditions. Lastly, examination of the adrenergic receptors responsible for initiating NE-mediated O_2_^●-^ production depicted a complex pattern of regulation. In CD4+ cells, the α2 receptor appeared to be primarily responsible for the NE-driven increase in O_2_^●-^, and this result agrees with a previous report of O_2_^●-^ production in human immune cells[19]. However, in CD8+ cells the pattern of adrenergic receptor regulation was complex and appeared to involve all receptors analyzed. It is unclear at this time why and how these patterns are divergent, but may be due to differential receptor density, sensitivity, or even mitochondrial content and signaling[75] between CD4+ and CD8+ T-lymphocytes. Investigations into these differences and their responses to adrenergic stimulation and blockade are highly warranted as patients currently taking adrenergic inhibitors may be subject to altered immune regulation[76] due to disproportionate binding of physiological NE concentrations to the remaining available adrenergic receptors.

In conclusion, our results present the unique observation that NE modulates T-lymphocyte function via alterations in mitochondrial metabolism and redox status, primarily through the generation of mitochondrial O_2_^●-^. The interplay of the nervous and immune systems is incredibly complex, and this study illuminates a single possible mechanism of regulation. It remains unclear how other neurotransmitters such as epinephrine, acetylcholine, neuropeptide Y, or substance P affect T-lymphocyte redox signaling. Moreover, the understanding of how these compounds affect the oxidative status of other components of the immune system (*i*.*e*. B-lymphocytes, macrophages, dendritic cells, etc.) remains unknown. The understanding of how these neural components dictate immune function may prove to be essential in the understanding of diseases with increased autonomic activity, and thus provide novel avenues for therapeutic intervention.

## Supporting Information

S1 Fig NE invokes a dose-dependent decrease in T-lymphocyte proliferation. T-lymphocytes were isolated, purified, and activated via CD3/CD28 stimulation in the presence of increasing amounts of NE. **A**. T-lymphocyte growth curves at various time points of *ex vivo* culture. N = 4. **B**. Representative flow cytometry histogram demonstrating population shift of NE-treated T-lymphocytes. *p<0.05 vs. 0 μM NE by Student’s t-test at respective time points.
(TIF)

S2 Fig NE increases O_2_^●-^ in all T-lymphocytes independent of activation stage. T-lymphocytes were isolated, purified, and activated via CD3/CD28 stimulation in the presence of 1 μM NE. **A**. Representative gating strategy and flow cytometry histograms to assess DHE oxidation in the various activation stages of T-lymphocytes at 96 hours with and without NE. N = Naïve, CM = Central Memory, E/M = Effector/Effector Memory. **B**. Quantification of activation stages in CD4+ (left) and CD8+ (right) T-lymphocytes activated in the presence of NE for 96 hours. N = 4. **C**. Quantification of DHE oxidation in various activation stages of CD4+ (left) and CD8+ (right) T-lymphocytes activated in the presence of NE for 96 hours. N = 4. *p<0.05 vs. 0 μM NE by 2-way ANOVA followed by Bonferroni post-hoc analysis.
(TIF)

S3 Fig NE does not directly oxidize DHE. NE was incubated in T-lymphocyte culture media in a cell free environment in the presence/absence of DHE for 30 minutes at 37°C. Quantification of fluorescence by spectrophotometry at the end of the assay demonstrating NE does not directly oxidize DHE in the absence of cells. N = 3. *p<0.05 vs. -DHE by 2-way ANOVA followed by Bonferroni post-hoc analysis.
(TIF)

S4 Fig Atomoxetine (Atom) inhibits T-lymphocyte proliferation independent of exogenous NE. T-lymphocytes were isolated, purified, and activated via CD3/CD28 stimulation in the presence of increasing amounts of the NE transport inhibitor Atom. **A.** T-lymphocyte growth curves at various time points of *ex vivo* culture. N = 5. **B**. T-lymphocyte growth curves at various time points of *ex vivo* culture in combination with NE. N = 5. *p<0.05 vs. 0 μM Atom (vehicle) by Student’s t-test at respective time points.
(TIF)

S5 Fig High doses of adrenergic antagonists inhibit CD4+ T-lymphocyte proliferation. CD4+ T-lymphocytes were isolated, purified, and activated via CD3/CD28 stimulation in the presence of increasing amounts of a respective adrenergic antagonist. T-lymphocyte growth curves at various time points of *ex vivo* culture with increasing amounts of **A**. prazosin (Praz; α1), **B**. atipamezole (Atip; α2), **C**. metoprolol (Met; β1), or **D**. ICI 118,511 (ICI; β2). N = 4. *p<0.05 vs. 0 μM (vehicle) by Student’s t-test at respective time points.
(TIF)

S6 Fig CD8+ T-lymphocytes show sensitivity to high dose adrenergic antagonists. CD8+ T-lymphocytes were isolated, purified, and activated via CD3/CD28 stimulation in the presence of increasing amounts of a respective adrenergic antagonist. T-lymphocyte growth curves at various time points of *ex vivo* culture with increasing amounts of **A**. prazosin (Praz; α1), **B**. atipamezole (Atip; α2), **C**. metoprolol (Met; β1), or **D**. ICI 118,511 (ICI; β2). N = 4. *p<0.05 vs. 0 μM (vehicle) by Student’s t-test at respective time points.
(TIF)

S7 Fig β-blockade rescues from NE-mediated decreases in proliferation. T-lymphocytes were isolated, purified, and activated via CD3/CD28 stimulation in the presence of 0 μM (vehicle) or 1 μM NE with 1 μM of the respective adrenergic antagonist (or combination). **A**. Upper, CD4+ T-lymphocyte growth curves at various time points of *ex vivo* culture with prazosin (Praz; α1) and/or atipamezole (Atip; α2). Lower, CD4+ T-lymphocyte growth curves at various time points of *ex vivo* culture with metoprolol (Met; β1) and/or ICI 118,511 (ICI; β2). **B.** Upper, CD8+ T-lymphocyte growth curves at various time points of *ex vivo* culture with prazosin (Praz; α1) and/or atipamezole (Atip; α2). Lower, CD8+ T-lymphocyte growth curves at various time points of *ex vivo* culture with metoprolol (Met; β1) and/or ICI 118,511 (ICI; β2). N = 4. *p<0.05 vs. 0 μM (vehicle), ^†^p<0.05 vs. NE by Student’s t-test at respective time points.
(TIF)

## References

[1] Guyenet PG. The sympathetic control of blood pressure. Nature reviews Neuroscience. 2006;7(5):335–46. Epub 2006/06/09. 10.1038/nrn1902 . 16760914

[2] Zucker IH. Novel mechanisms of sympathetic regulation in chronic heart failure. Hypertension. 2006;48(6):1005–11. 10.1161/01.HYP.0000246614.47231.25 17015773

[3] Iyngkaran P, Anavekar N, Majoni W, Thomas MC. The role and management of sympathetic overactivity in cardiovascular and renal complications of diabetes. Diabetes & metabolism. 2013;39(4):290–8. Epub 2013/07/23. 10.1016/j.diabet.2013.05.002 . 23871308

[4] Carter JR, Goldstein DS. Sympathoneural and adrenomedullary responses to mental stress. Comprehensive Physiology. 2015;5(1):119–46. Epub 2015/01/16. 10.1002/cphy.c140030 . 25589266 PMC5280073

[5] Nance DM, Sanders VM. Autonomic innervation and regulation of the immune system (1987–2007). Brain, behavior, and immunity. 2007;21(6):736–45. Epub 2007/05/01. 10.1016/j.bbi.2007.03.008 17467231 PMC1986730

[6] Sanders VM. The role of adrenoceptor-mediated signals in the modulation of lymphocyte function. Advances in neuroimmunology. 1995;5(3):283–98. Epub 1995/01/01. 10.1016/0960-5428(95)00019-X . 8748072

[7] Maestroni GJ. Adrenergic modulation of dendritic cells function: relevance for the immune homeostasis. Current neurovascular research. 2005;2(2):169–73. Epub 2005/09/27. 10.2174/1567202053586776 . 16181110

[8] Kenney MJ, Ganta CK. Autonomic nervous system and immune system interactions. Comprehensive Physiology. 2014;4(3):1177–200. Epub 2014/06/20. 10.1002/cphy.c130051 24944034 PMC4374437

[9] Padro CJ, Sanders VM. Neuroendocrine regulation of inflammation. Seminars in immunology. 2014;26(5):357–68. Epub 2014/02/04. 10.1016/j.smim.2014.01.003 24486056 PMC4116469

[10] Case AJ, Zimmerman MC. Sympathetic-mediated activation versus suppression of the immune system: consequences for hypertension. The Journal of physiology. 2016;594(3):527–36. Epub 2016/02/03. 10.1113/JP271516 . 26830047 PMC4930069

[11] Strell C, Sievers A, Bastian P, Lang K, Niggemann B, Zanker KS, et al Divergent effects of norepinephrine, dopamine and substance P on the activation, differentiation and effector functions of human cytotoxic T lymphocytes. BMC immunology. 2009;10:62 Epub 2009/12/09. 10.1186/1471-2172-10-62 19968887 PMC2794263

[12] Chouaib S, Welte K, Dupont B. Differential effect of anti-beta 2-microglobulin on IL 2 production and IL 2 receptor expression in the primary mixed lymphocyte culture reaction. Journal of immunology. 1985;134(2):940–8. Epub 1985/02/01. . 3155544

[13] Novak TJ, Rothenberg EV. cAMP inhibits induction of interleukin 2 but not of interleukin 4 in T cells. Proc Natl Acad Sci U S A. 1990;87(23):9353–7. Epub 1990/12/01. 10.1073/pnas.87.23.9353 2174560 PMC55163

[14] Kohm AP, Sanders VM. Norepinephrine and beta 2-adrenergic receptor stimulation regulate CD4+ T and B lymphocyte function in vitro and in vivo. Pharmacological reviews. 2001;53(4):487–525. Epub 2001/12/06. . 11734616

[15] Takayanagi Y, Osawa S, Ikuma M, Takagaki K, Zhang J, Hamaya Y, et al Norepinephrine suppresses IFN-gamma and TNF-alpha production by murine intestinal intraepithelial lymphocytes via the beta(1) adrenoceptor. Journal of neuroimmunology. 2012;245(1–2):66–74. Epub 2012/03/09. 10.1016/j.jneuroim.2012.02.007 . 22398028

[16] Madden KS, Moynihan JA, Brenner GJ, Felten SY, Felten DL, Livnat S. Sympathetic nervous system modulation of the immune system. III. Alterations in T and B cell proliferation and differentiation in vitro following chemical sympathectomy. Journal of neuroimmunology. 1994;49(1–2):77–87. Epub 1994/01/01. 10.1016/0165-5728(94)90183-X . 8294564

[17] Swanson MA, Lee WT, Sanders VM. IFN-gamma production by Th1 cells generated from naive CD4+ T cells exposed to norepinephrine. Journal of immunology. 2001;166(1):232–40. Epub 2000/12/21. . 11123297 10.4049/jimmunol.166.1.232

[18] Alaniz RC, Thomas SA, Perez-Melgosa M, Mueller K, Farr AG, Palmiter RD, et al Dopamine beta-hydroxylase deficiency impairs cellular immunity. Proc Natl Acad Sci U S A. 1999;96(5):2274–8. Epub 1999/03/03. 10051631 10.1073/pnas.96.5.2274 PMC26773

[19] Deo SH, Jenkins NT, Padilla J, Parrish AR, Fadel PJ. Norepinephrine increases NADPH oxidase-derived superoxide in human peripheral blood mononuclear cells via alpha-adrenergic receptors. American journal of physiology Regulatory, integrative and comparative physiology. 2013;305(10):R1124–32. Epub 2013/09/27. 10.1152/ajpregu.00347.2013 24068047 PMC3841802

[20] Livnat S, Madden KS, Felten DL, Felten SY. Regulation of the immune system by sympathetic neural mechanisms. Progress in neuro-psychopharmacology & biological psychiatry. 1987;11(2–3):145–52. Epub 1987/01/01. 10.1016/0278-5846(87)90052-2 . 2819949

[21] Huang HW, Fang XX, Wang XQ, Peng YP, Qiu YH. Regulation of differentiation and function of helper T cells by lymphocyte-derived catecholamines via alpha(1)- and beta(2)-adrenoceptors. Neuroimmunomodulation. 2015;22(3):138–51. Epub 2014/05/08. 10.1159/000360579 . 24800755

[22] Case AJ, Zimmerman MC. Redox-regulated suppression of splenic T-lymphocyte activation in a model of sympathoexcitation. Hypertension. 2015;65(4):916–23. Epub 2015/02/19. 10.1161/HYPERTENSIONAHA.114.05075 25691620 PMC4359089

[23] Sena LA, Li S, Jairaman A, Prakriya M, Ezponda T, Hildeman DA, et al Mitochondria are required for antigen-specific T cell activation through reactive oxygen species signaling. Immunity. 2013;38(2):225–36. Epub 2013/02/19. 10.1016/j.immuni.2012.10.020 23415911 PMC3582741

[24] Case AJ, Madsen JM, Motto DG, Meyerholz DK, Domann FE. Manganese superoxide dismutase depletion in murine hematopoietic stem cells perturbs iron homeostasis, globin switching, and epigenetic control in erythrocyte precursor cells. Free Radic Biol Med. 2013;56:17–27. Epub 2012/12/12. 10.1016/j.freeradbiomed.2012.11.018 23219873 PMC3578015

[25] Case AJ, McGill JL, Tygrett LT, Shirasawa T, Spitz DR, Waldschmidt TJ, et al Elevated mitochondrial superoxide disrupts normal T cell development, impairing adaptive immune responses to an influenza challenge. Free Radic Biol Med. 2011;50(3):448–58. Epub 2010/12/07. 10.1016/j.freeradbiomed.2010.11.025 21130157 PMC3026081

[26] O'Sullivan D, Pearce EL. Immunology. Expanding the role of metabolism in T cells. Science. 2015;348(6238):976–7. Epub 2015/05/30. 10.1126/science.aac4997 . 26023125

[27] Pearce EL, Pearce EJ. Metabolic pathways in immune cell activation and quiescence. Immunity. 2013;38(4):633–43. Epub 2013/04/23. 10.1016/j.immuni.2013.04.005 23601682 PMC3654249

[28] Pearce EL, Poffenberger MC, Chang CH, Jones RG. Fueling immunity: insights into metabolism and lymphocyte function. Science. 2013;342(6155):1242454 Epub 2013/10/12. 10.1126/science.1242454 24115444 PMC4486656

[29] Landi MS, Kreider JW, Lang CM, Bullock LP. Effects of shipping on the immune function in mice. American journal of veterinary research. 1982;43(9):1654–7. Epub 1982/09/01. . 7149414

[30] Olfe J, Domanska G, Schuett C, Kiank C. Different stress-related phenotypes of BALB/c mice from in-house or vendor: alterations of the sympathetic and HPA axis responsiveness. BMC physiology. 2010;10:2 Epub 2010/03/11. 10.1186/1472-6793-10-2 20214799 PMC2845127

[31] Sorge RE, Martin LJ, Isbester KA, Sotocinal SG, Rosen S, Tuttle AH, et al Olfactory exposure to males, including men, causes stress and related analgesia in rodents. Nature methods. 2014;11(6):629–32. Epub 2014/04/30. 10.1038/nmeth.2935 . 24776635

[32] Qiu YH, Cheng C, Dai L, Peng YP. Effect of endogenous catecholamines in lymphocytes on lymphocyte function. Journal of neuroimmunology. 2005;167(1–2):45–52. Epub 2005/07/06. 10.1016/j.jneuroim.2005.06.007 . 15996757

[33] Ramer-Quinn DS, Swanson MA, Lee WT, Sanders VM. Cytokine production by naive and primary effector CD4+ T cells exposed to norepinephrine. Brain, behavior, and immunity. 2000;14(4):239–55. Epub 2000/12/20. 10.1006/brbi.2000.0603 . 11120594

[34] Slota C, Shi A, Chen G, Bevans M, Weng NP. Norepinephrine preferentially modulates memory CD8 T cell function inducing inflammatory cytokine production and reducing proliferation in response to activation. Brain, behavior, and immunity. 2015;46:168–79. Epub 2015/02/06. 10.1016/j.bbi.2015.01.015 25653192 PMC4414741

[35] Wahle M, Hanefeld G, Brunn S, Straub RH, Wagner U, Krause A, et al Failure of catecholamines to shift T-cell cytokine responses toward a Th2 profile in patients with rheumatoid arthritis. Arthritis research & therapy. 2006;8(5):R138 Epub 2006/08/08. 10.1186/ar2028 16889669 PMC1779439

[36] Case AJ, Li S, Basu U, Tian J, Zimmerman MC. Mitochondrial-localized NADPH oxidase 4 is a source of superoxide in angiotensin II-stimulated neurons. American journal of physiology Heart and circulatory physiology. 2013;305(1):H19–28. Epub 2013/04/30. 10.1152/ajpheart.00974.2012 23624625 PMC3727106

[37] Sanders VM. The beta2-adrenergic receptor on T and B lymphocytes: do we understand it yet? Brain, behavior, and immunity. 2012;26(2):195–200. Epub 2011/08/23. 10.1016/j.bbi.2011.08.001 21855626 PMC3243812

[38] Yamazaki I, Mason HS, Piette L. Identification, by electron paramagnetic resonance spectroscopy, of free radicals generated from substrates by peroxidase. J Biol Chem. 1960;235:2444–9. Epub 1960/08/01. . 13846434

[39] Mason HS, Spencer E, Yamazaki I. Identification by electron spin resonance spectroscopy of the primary product of tyrosinase-catalyzed catechol oxidation. Biochemical and biophysical research communications. 1961;4:236–8. Epub 1961/03/10. 10.1016/0006-291X(61)90278-9 . 13767818

[40] Kalyanaraman B, Felix CC, Sealy RC. Peroxidatic oxidation of catecholamines. A kinetic electron spin resonance investigation using the spin stabilization approach. J Biol Chem. 1984;259(12):7584–9. Epub 1984/06/25. . 6330064

[41] Zimmerman MC, Dunlay RP, Lazartigues E, Zhang Y, Sharma RV, Engelhardt JF, et al Requirement for Rac1-dependent NADPH oxidase in the cardiovascular and dipsogenic actions of angiotensin II in the brain. CircRes. 2004;95(5):532–9. 10.1161/01.RES.0000139957.22530.b9 15271858

[42] Bedard K, Krause KH. The NOX family of ROS-generating NADPH oxidases: physiology and pathophysiology. Physiol Rev. 2007;87(1):245–313. 10.1152/physrev.00044.2005 17237347

[43] Drose S, Brandt U. Molecular mechanisms of superoxide production by the mitochondrial respiratory chain. Advances in experimental medicine and biology. 2012;748:145–69. Epub 2012/06/26. 10.1007/978-1-4614-3573-0_6 . 22729857

[44] Zorov DB, Juhaszova M, Sollott SJ. Mitochondrial reactive oxygen species (ROS) and ROS-induced ROS release. Physiol Rev. 2014;94(3):909–50. Epub 2014/07/06. 10.1152/physrev.00026.2013 24987008 PMC4101632

[45] Ferrick DA, Neilson A, Beeson C. Advances in measuring cellular bioenergetics using extracellular flux. Drug discovery today. 2008;13(5–6):268–74. Epub 2008/03/18. 10.1016/j.drudis.2007.12.008 . 18342804

[46] Sansbury BE, Jones SP, Riggs DW, Darley-Usmar VM, Hill BG. Bioenergetic function in cardiovascular cells: the importance of the reserve capacity and its biological regulation. Chemico-biological interactions. 2011;191(1–3):288–95. Epub 2010/12/15. 10.1016/j.cbi.2010.12.002 21147079 PMC3090710

[47] Yadava N, Nicholls DG. Spare respiratory capacity rather than oxidative stress regulates glutamate excitotoxicity after partial respiratory inhibition of mitochondrial complex I with rotenone. The Journal of neuroscience: the official journal of the Society for Neuroscience. 2007;27(27):7310–7. Epub 2007/07/06. 10.1523/JNEUROSCI.0212-07.2007 . 17611283 PMC6794596

[48] Nicholls DG. Oxidative stress and energy crises in neuronal dysfunction. Ann N Y Acad Sci. 2008;1147:53–60. Epub 2008/12/17. 10.1196/annals.1427.002 . 19076430

[49] Dranka BP, Hill BG, Darley-Usmar VM. Mitochondrial reserve capacity in endothelial cells: The impact of nitric oxide and reactive oxygen species. Free Radic Biol Med. 2010;48(7):905–14. Epub 2010/01/23. 10.1016/j.freeradbiomed.2010.01.015 20093177 PMC2860730

[50] Faraj BA, Olkowski ZL, Jackson RT. Expression of a high-affinity serotonin transporter in human lymphocytes. International journal of immunopharmacology. 1994;16(7):561–7. Epub 1994/07/01. 10.1016/0192-0561(94)90107-4 . 7928004

[51] Gordon J, Barnes NM. Lymphocytes transport serotonin and dopamine: agony or ecstasy? Trends in immunology. 2003;24(8):438–43. Epub 2003/08/12. 10.1016/S1471-4906(03)00176-5 . 12909457

[52] Mata S, Urbina M, Manzano E, Ortiz T, Lima L. Noradrenaline transporter and its turnover rate are decreased in blood lymphocytes of patients with major depression. Journal of neuroimmunology. 2005;170(1–2):134–40. Epub 2005/10/26. 10.1016/j.jneuroim.2005.08.007 . 16242784

[53] Qiu YH, Peng YP, Jiang JM, Wang JJ. Expression of tyrosine hydroxylase in lymphocytes and effect of endogenous catecholamines on lymphocyte function. Neuroimmunomodulation. 2004;11(2):75–83. Epub 2004/02/06. 10.1159/000075316 . 14758053

[54] Devadas S, Zaritskaya L, Rhee SG, Oberley L, Williams MS. Discrete generation of superoxide and hydrogen peroxide by T cell receptor stimulation: selective regulation of mitogen-activated protein kinase activation and fas ligand expression. The Journal of experimental medicine. 2002;195(1):59–70. Epub 2002/01/10. 10.1084/jem.20010659 11781366 PMC2196010

[55] Jackson SH, Devadas S, Kwon J, Pinto LA, Williams MS. T cells express a phagocyte-type NADPH oxidase that is activated after T cell receptor stimulation. Nat Immunol. 2004;5(8):818–27. Epub 2004/07/20. 10.1038/ni1096 . 15258578

[56] Jackson EK. The 2',3'-cAMP-adenosine pathway. American journal of physiology Renal physiology. 2011;301(6):F1160–7. Epub 2011/09/23. 10.1152/ajprenal.00450.2011 21937608 PMC3233866

[57] Azarashvili T, Krestinina O, Galvita A, Grachev D, Baburina Y, Stricker R, et al Ca2+-dependent permeability transition regulation in rat brain mitochondria by 2',3'-cyclic nucleotides and 2',3'-cyclic nucleotide 3'-phosphodiesterase. American journal of physiology Cell physiology. 2009;296(6):C1428–39. Epub 2009/04/10. 10.1152/ajpcell.00006.2009 . 19357238

[58] Buck MD, O'Sullivan D, Pearce EL. T cell metabolism drives immunity. The Journal of experimental medicine. 2015;212(9):1345–60. Epub 2015/08/12. 10.1084/jem.20151159 26261266 PMC4548052

[59] O'Sullivan D, van der Windt GJ, Huang SC, Curtis JD, Chang CH, Buck MD, et al Memory CD8(+) T cells use cell-intrinsic lipolysis to support the metabolic programming necessary for development. Immunity. 2014;41(1):75–88. Epub 2014/07/09. 10.1016/j.immuni.2014.06.005 25001241 PMC4120664

[60] Pearce EL, Walsh MC, Cejas PJ, Harms GM, Shen H, Wang LS, et al Enhancing CD8 T-cell memory by modulating fatty acid metabolism. Nature. 2009;460(7251):103–7. Epub 2009/06/06. 10.1038/nature08097 19494812 PMC2803086

[61] van der Windt GJ, Everts B, Chang CH, Curtis JD, Freitas TC, Amiel E, et al Mitochondrial respiratory capacity is a critical regulator of CD8+ T cell memory development. Immunity. 2012;36(1):68–78. Epub 2011/12/31. 10.1016/j.immuni.2011.12.007 22206904 PMC3269311

[62] van der Windt GJ, O'Sullivan D, Everts B, Huang SC, Buck MD, Curtis JD, et al CD8 memory T cells have a bioenergetic advantage that underlies their rapid recall ability. Proc Natl Acad Sci U S A. 2013;110(35):14336–41. Epub 2013/08/14. 10.1073/pnas.1221740110 23940348 PMC3761631

[63] van der Windt GJ, Pearce EL. Metabolic switching and fuel choice during T-cell differentiation and memory development. Immunological reviews. 2012;249(1):27–42. Epub 2012/08/15. 10.1111/j.1600-065X.2012.01150.x 22889213 PMC3645891

[64] Kin NW, Sanders VM. It takes nerve to tell T and B cells what to do. Journal of leukocyte biology. 2006;79(6):1093–104. Epub 2006/03/15. 10.1189/jlb.1105625 . 16531560

[65] Chang CH, Curtis JD, Maggi LB Jr., Faubert B, Villarino AV, O'Sullivan D, et al Posttranscriptional control of T cell effector function by aerobic glycolysis. Cell. 2013;153(6):1239–51. Epub 2013/06/12. 10.1016/j.cell.2013.05.016 23746840 PMC3804311

[66] Bi Y, Yang R. Direct and indirect regulatory mechanisms in TH17 cell differentiation and functions. Scandinavian journal of immunology. 2012;75(6):543–52. Epub 2012/01/21. 10.1111/j.1365-3083.2012.02686.x . 22260240

[67] Kleinewietfeld M, Manzel A, Titze J, Kvakan H, Yosef N, Linker RA, et al Sodium chloride drives autoimmune disease by the induction of pathogenic TH17 cells. Nature. 2013;496(7446):518–22. Epub 2013/03/08. 10.1038/nature11868 23467095 PMC3746493

[68] Harrison DG, Guzik TJ, Lob HE, Madhur MS, Marvar PJ, Thabet SR, et al Inflammation, immunity, and hypertension. Hypertension. 2011;57(2):132–40. 10.1161/HYPERTENSIONAHA.110.163576 21149826 PMC3028593

[69] O'Donovan A, Cohen BE, Seal KH, Bertenthal D, Margaretten M, Nishimi K, et al Elevated risk for autoimmune disorders in iraq and afghanistan veterans with posttraumatic stress disorder. Biological psychiatry. 2015;77(4):365–74. Epub 2014/08/12. 10.1016/j.biopsych.2014.06.015 25104173 PMC4277929

[70] Lee YC, Agnew-Blais J, Malspeis S, Keyes K, Costenbader K, Kubzansky LD, et al Posttraumatic Stress Disorder and Risk for Incident Rheumatoid Arthritis. Arthritis care & research. 2015 Epub 2015/08/05. 10.1002/acr.22683 26239524 PMC4740283

[71] Mikuls TR, Padala PR, Sayles HR, Yu F, Michaud K, Caplan L, et al Prospective study of posttraumatic stress disorder and disease activity outcomes in US veterans with rheumatoid arthritis. Arthritis care & research. 2013;65(2):227–34. Epub 2012/06/29. 10.1002/acr.21778 . 22740431

[72] Krystal JH, Neumeister A. Noradrenergic and serotonergic mechanisms in the neurobiology of posttraumatic stress disorder and resilience. Brain Res. 2009;1293:13–23. Epub 2009/04/01. 10.1016/j.brainres.2009.03.044 19332037 PMC2761677

[73] Palm S, Hinrichsen H, Barth J, Halabi A, Ferstl R, Tolk J, et al Modulation of lymphocyte subsets due to psychological stress in patients with rheumatoid arthritis. Eur J Clin Invest. 1992;22 Suppl 1:26–9. Epub 1992/10/01. . 1459183

[74] Baerwald CG, Wahle M, Ulrichs T, Jonas D, von Bierbrauer A, von Wichert P, et al Reduced catecholamine response of lymphocytes from patients with rheumatoid arthritis. Immunobiology. 1999;200(1):77–91. Epub 1999/03/20. . 10084697 10.1016/s0171-2985(99)80034-5

[75] Ma C, Kesarwala AH, Eggert T, Medina-Echeverz J, Kleiner DE, Jin P, et al NAFLD causes selective CD4(+) T lymphocyte loss and promotes hepatocarcinogenesis. Nature. 2016;531(7593):253–7. Epub 2016/03/05. 10.1038/nature16969 26934227 PMC4786464

[76] Group PS, Devereaux PJ, Yang H, Yusuf S, Guyatt G, Leslie K, et al Effects of extended-release metoprolol succinate in patients undergoing non-cardiac surgery (POISE trial): a randomised controlled trial. Lancet. 2008;371(9627):1839–47. Epub 2008/05/16. 10.1016/S0140-6736(08)60601-7 . 18479744

