# Supplementary figures and images for: Mitochondrial Superoxide Signaling Contributes to Norepinephrine-Mediated T-Lymphocyte Cytokine Profiles
Source: PLoS One. 2016 Oct 11;11(10):e0164609. doi: 10.1371/journal.pone.0164609 (PMC5058488; doi:10.1371/journal.pone.0164609)

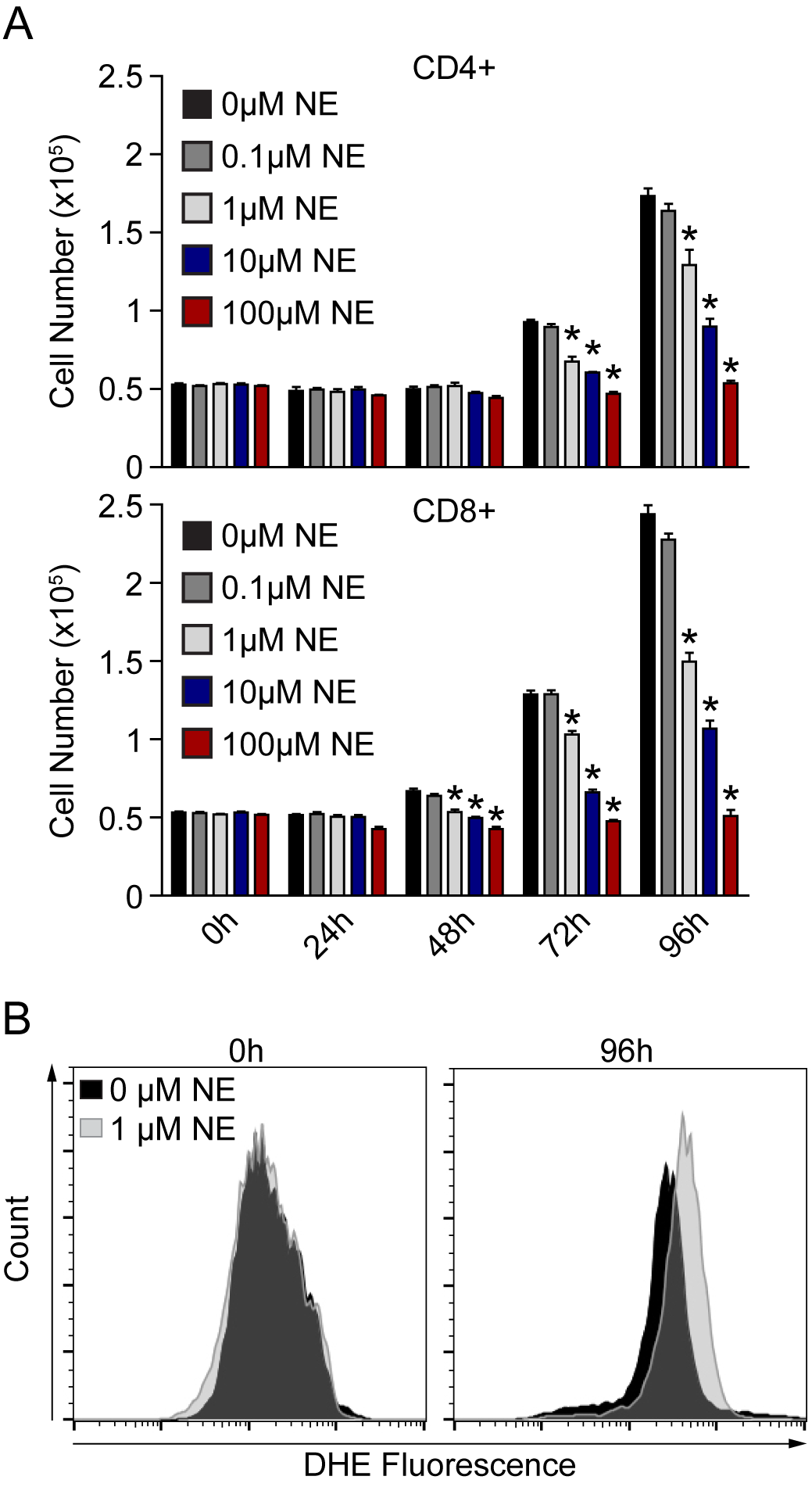

Supplement: S1 Fig — T-lymphocytes were isolated, purified, and activated via CD3/CD28 stimulation in the presence of increasing amounts of NE. A. T-lymphocyte growth curves at various time points of ex vivo culture. N = 4. B. Representative flow cytometry histogram demonstrating population shift of NE-treated T-lymphocytes. *p<0.05 vs. 0 μM NE by Student’s t-test at respective time points. (TIF) [file pone.0164609.s001.tif]

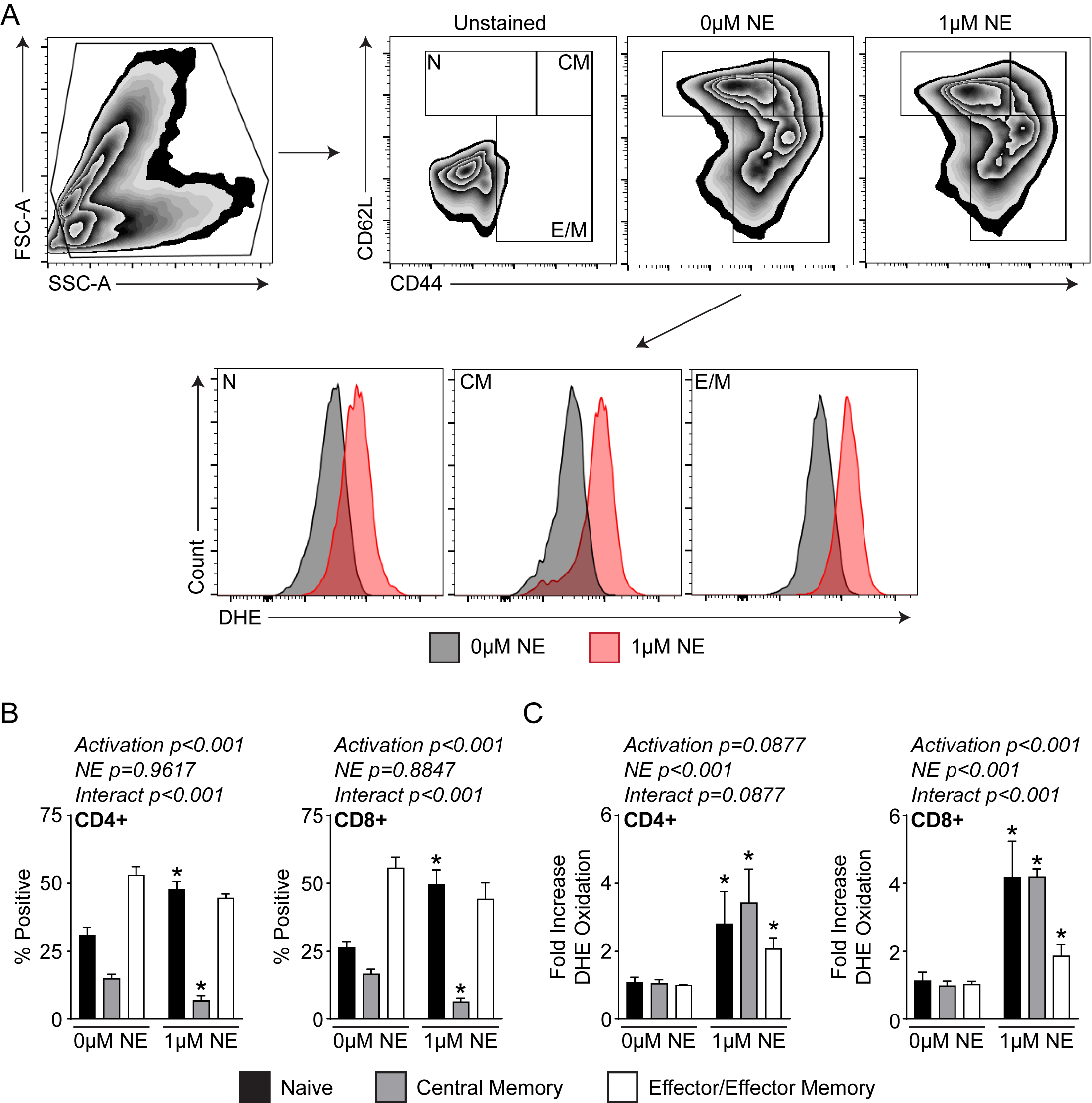

Supplement: S2 Fig — T-lymphocytes were isolated, purified, and activated via CD3/CD28 stimulation in the presence of 1 μM NE. A. Representative gating strategy and flow cytometry histograms to assess DHE oxidation in the various activation stages of T-lymphocytes at 96 hours with and without NE. N = Naïve, CM = Central Memory, E/M = Effector/Effector Memory. B. Quantification of activation stages in CD4+ (left) and CD8+ (right) T-lymphocytes activated in the presence of NE for 96 hours. N = 4. C. Quantification of DHE oxidation in various activation stages of CD4+ (left) and CD8+ (right) T-lymphocytes activated in the presence of NE for 96 hours. N = 4. *p<0.05 vs. 0 μM NE by 2-way ANOVA followed by Bonferroni post-hoc analysis. (TIF) [file pone.0164609.s002.tif]

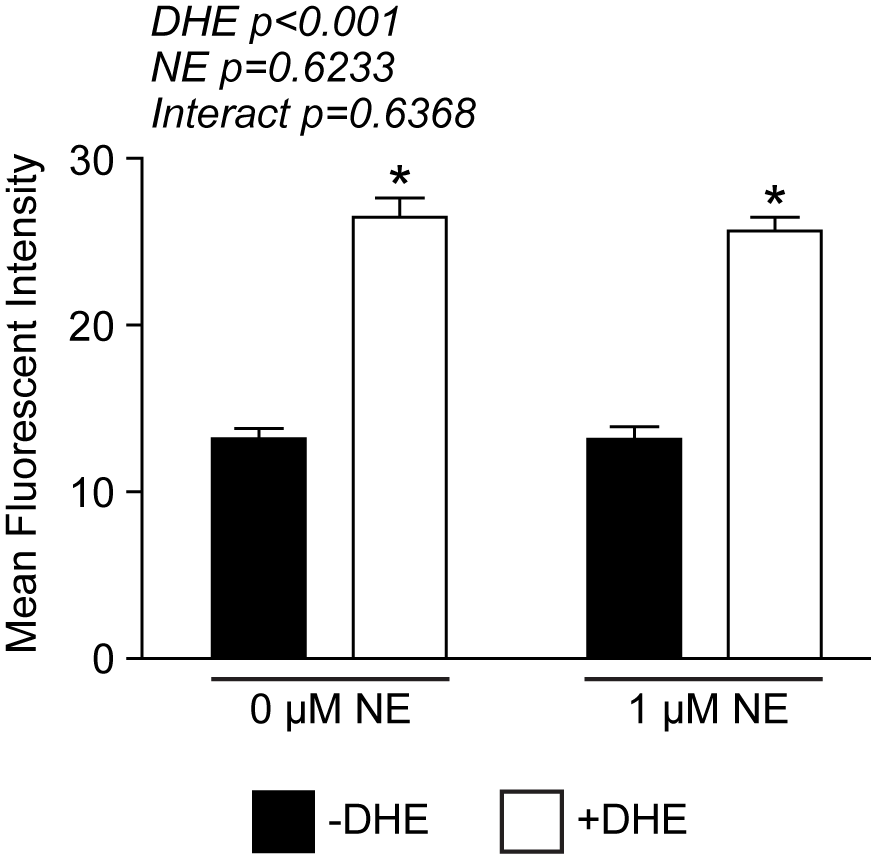

Supplement: S3 Fig — NE was incubated in T-lymphocyte culture media in a cell free environment in the presence/absence of DHE for 30 minutes at 37°C. Quantification of fluorescence by spectrophotometry at the end of the assay demonstrating NE does not directly oxidize DHE in the absence of cells. N = 3. *p<0.05 vs. -DHE by 2-way ANOVA followed by Bonferroni post-hoc analysis. (TIF) [file pone.0164609.s003.tif]

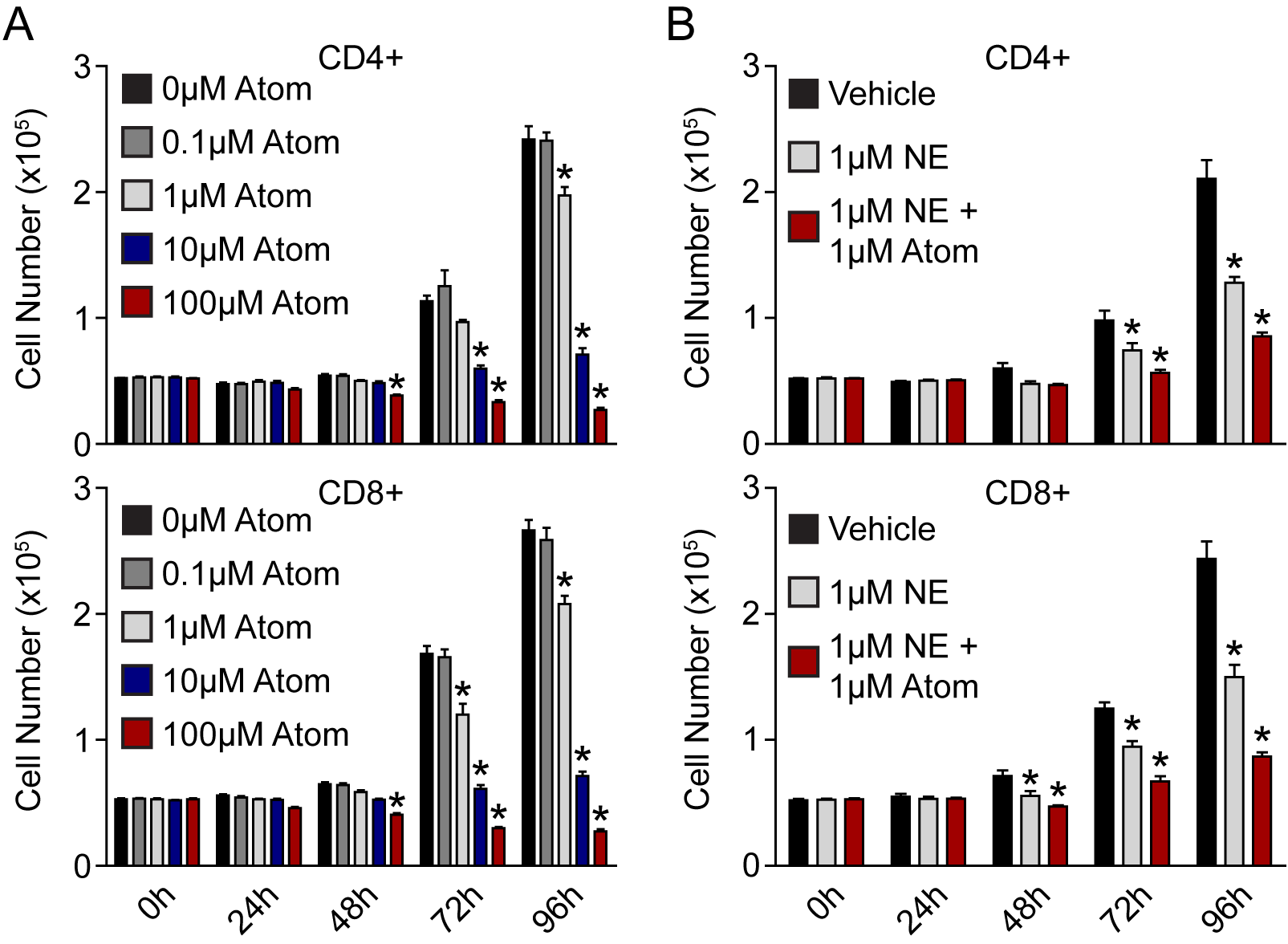

Supplement: S4 Fig — T-lymphocytes were isolated, purified, and activated via CD3/CD28 stimulation in the presence of increasing amounts of the NE transport inhibitor Atom. A. T-lymphocyte growth curves at various time points of ex vivo culture. N = 5. B. T-lymphocyte growth curves at various time points of ex vivo culture in combination with NE. N = 5. *p<0.05 vs. 0 μM Atom (vehicle) by Student’s t-test at respective time points. (TIF) [file pone.0164609.s004.tif]

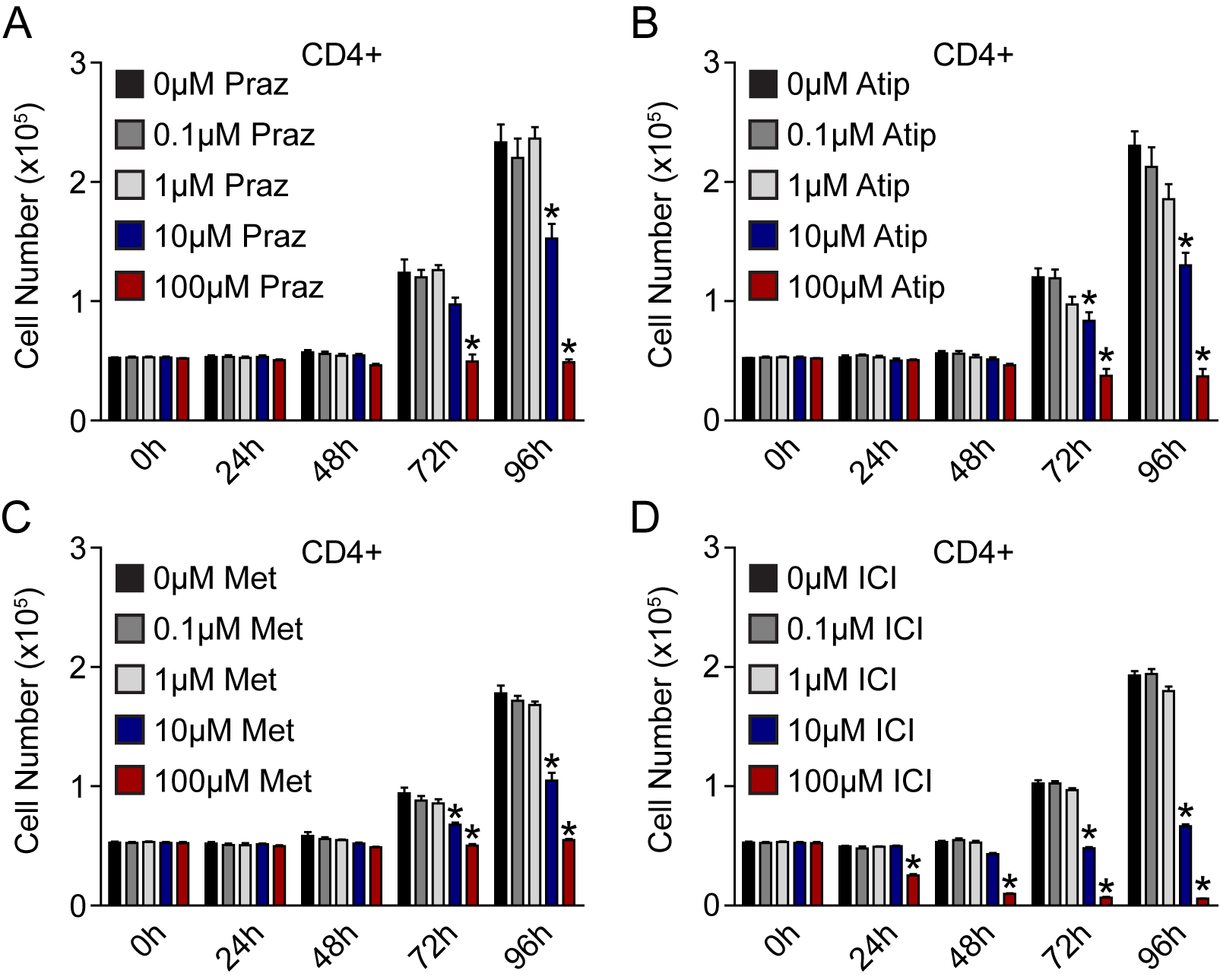

Supplement: S5 Fig — CD4+ T-lymphocytes were isolated, purified, and activated via CD3/CD28 stimulation in the presence of increasing amounts of a respective adrenergic antagonist. T-lymphocyte growth curves at various time points of ex vivo culture with increasing amounts of A. prazosin (Praz; α1), B. atipamezole (Atip; α2), C. metoprolol (Met; β1), or D. ICI 118,511 (ICI; β2). N = 4. *p<0.05 vs. 0 μM (vehicle) by Student’s t-test at respective time points. (TIF) [file pone.0164609.s005.tif]

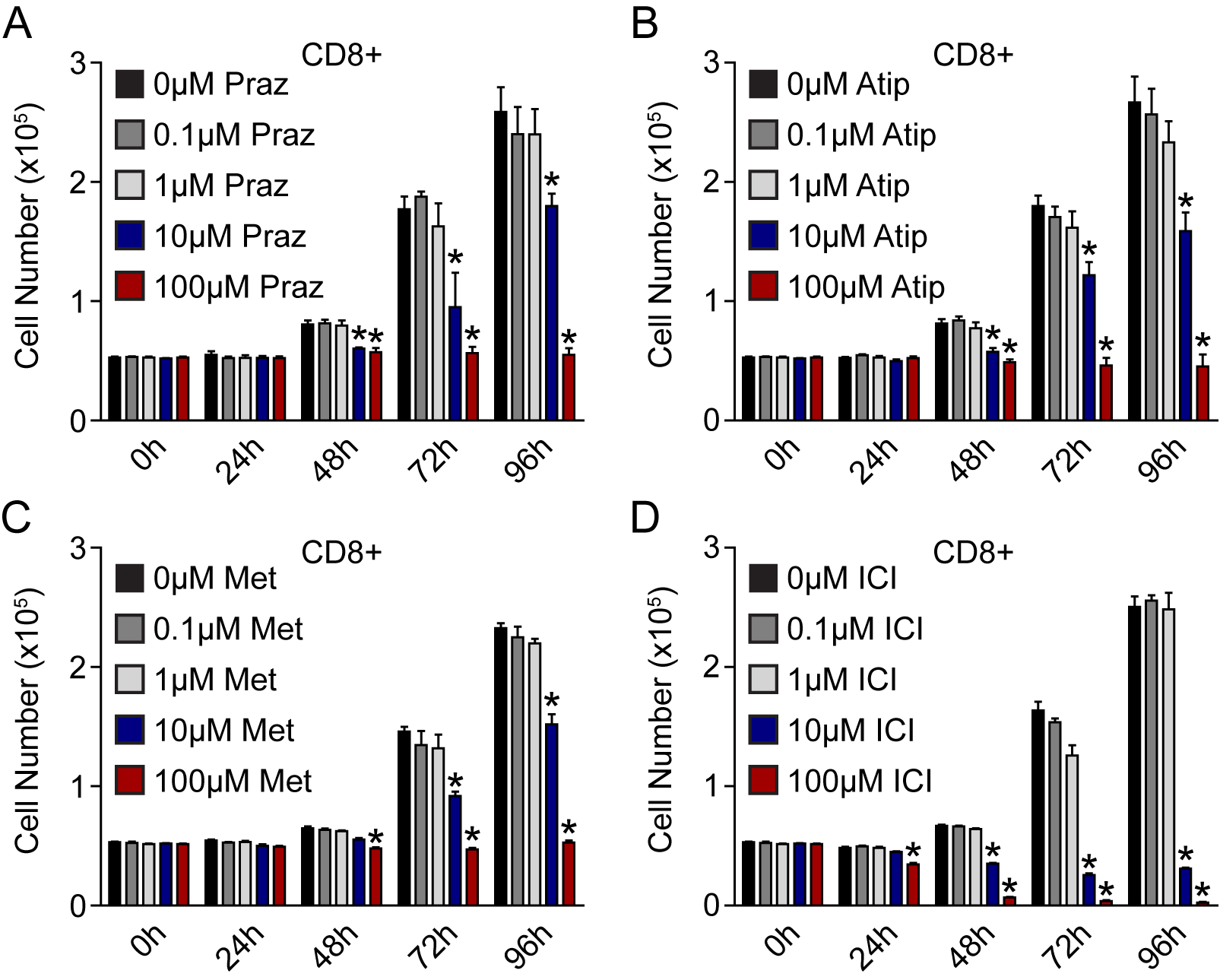

Supplement: S6 Fig — CD8+ T-lymphocytes were isolated, purified, and activated via CD3/CD28 stimulation in the presence of increasing amounts of a respective adrenergic antagonist. T-lymphocyte growth curves at various time points of ex vivo culture with increasing amounts of A. prazosin (Praz; α1), B. atipamezole (Atip; α2), C. metoprolol (Met; β1), or D. ICI 118,511 (ICI; β2). N = 4. *p<0.05 vs. 0 μM (vehicle) by Student’s t-test at respective time points. (TIF) [file pone.0164609.s006.tif]

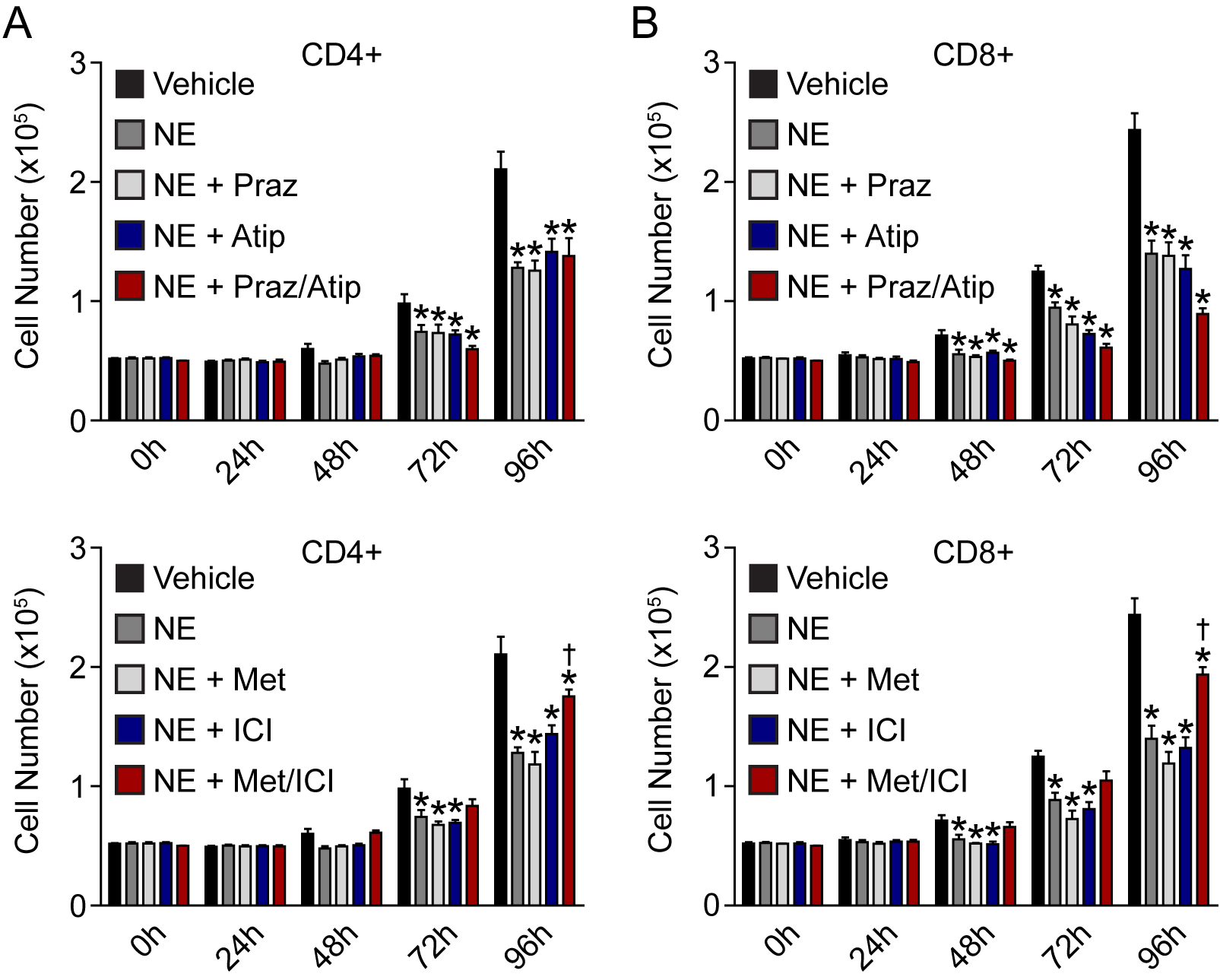

Supplement: S7 Fig — T-lymphocytes were isolated, purified, and activated via CD3/CD28 stimulation in the presence of 0 μM (vehicle) or 1 μM NE with 1 μM of the respective adrenergic antagonist (or combination). A. Upper, CD4+ T-lymphocyte growth curves at various time points of ex vivo culture with prazosin (Praz; α1) and/or atipamezole (Atip; α2). Lower, CD4+ T-lymphocyte growth curves at various time points of ex vivo culture with metoprolol (Met; β1) and/or ICI 118,511 (ICI; β2). B. Upper, CD8+ T-lymphocyte growth curves at various time points of ex vivo culture with prazosin (Praz; α1) and/or atipamezole (Atip; α2). Lower, CD8+ T-lymphocyte growth curves at various time points of ex vivo culture with metoprolol (Met; β1) and/or ICI 118,511 (ICI; β2). N = 4. *p<0.05 vs. 0 μM (vehicle), †p<0.05 vs. NE by Student’s t-test at respective time points. (TIF) [file pone.0164609.s007.tif]
